# Molecular pattern of lncRNAs in hepatocellular carcinoma

**DOI:** 10.1186/s13046-019-1213-0

**Published:** 2019-05-16

**Authors:** Haoming Mai, Bin Zhou, Li Liu, Fu Yang, Carly Conran, Yuan Ji, Jinlin Hou, Deke Jiang

**Affiliations:** 1 State Key Laboratory of Organ Failure Research, Guangdong Key Laboratory of Viral Hepatitis Research, Institute of Liver Diseases Research of Guangdong Province, Guangzhou, China; 2 Department of Infectious Diseases and Hepatology Unit, Nanfang Hospital, Southern Medical University, Guangzhou, 510515 China; 3 Department of Medical Genetics, Second Military Medical University, Shanghai, 200433 China; 4 University of Illinois College of Medicine, Chicago, IL 60612 USA; 5 Department of Public Health Sciences, University of Chicago, Chicago, IL 60637 USA

**Keywords:** lncRNA, Molecular pattern, Regulation of biological processes, Hepatocellular carcinoma, Well-characterized mechanism

## Abstract

Hepatocellular carcinoma (HCC) is one of the most notable lethal malignancies worldwide. However, the molecular mechanisms involved in the initiation and progression of this disease remain poorly understood. Over the past decade, many studies have demonstrated the important regulatory roles of long non-coding RNAs (lncRNAs) in HCC. Here, we comprehensively review recent discoveries regarding HCC-associated lncRNA functions, which we have classified and described according to their mechanism models.

**Electronic supplementary material:**

The online version of this article (10.1186/s13046-019-1213-0) contains supplementary material, which is available to authorized users.

## Background

According to the World Health Organization (WHO), liver cancer is the sixth most common malignancy worldwide. In 2018, there were approximately 841,000 new liver cancer patients in the world, and approximately 83% occurred in Eastern Asia. The prognosis of liver cancer is very poor, and the mortality rate is high, resulting in approximately 781,000 deaths in 2018, which is the fourth most common cause of cancer deaths (approximately 8.2% of cancer deaths). Hepatocellular carcinoma (HCC) is the primary form of liver cancer, comprising 75–85% of cases [1]. Prognosis is closely related to early diagnosis of the disease. However, HCC has a lengthy subclinical stage, with an insidious initiation and progression that is often difficult to detect. Thus, most HCC patients are diagnosed at an advanced stage, and treatment options at this stage are limited. Moreover, the development of HCC is a multi-step process involving many gene networks and changes in signaling pathways, and many of these pathways remain to be elucidated [2]. It is therefore important to decipher the molecular pathological mechanism of HCC to better assess patient prognosis and identify, or develop, optimal therapies.

According to the biological central dogma, RNAs are considered to be a template for protein translation (mRNAs) and the infrastructure supporting this process (tRNAs and rRNAs) [3]. However, knowledge gained from human genome sequencing has challenged this rule [4]. The number of “genes” estimated by express sequence tag data in the year 2000 ranged from 45,000 to 140,000 [5]. Later, the International Human Genome Sequencing Consortium proposed that the human genome has only 20,000 to 25,000 protein-coding genes [6]. Soon after, the ENCyclopedia Of DNA Elements (ENCODE) project noted that only 2% of the human genome is encoded into protein, though 74.7% of the human genome is transcribed, with no cell line expressing more than 56.7% of this human transcriptome collection [7]. Furthermore, 62% of the human genome encodes long RNA molecules (> 200 nucleotides) [8]. These pervasive studies brought to light the functional importance of this so-called “junk” DNA. Non-coding RNAs (ncRNAs) are not just “transcriptional noise,” as previously thought, which fundamentally changes our interpretation of the genome and transcriptome [9, 10].

The development of high-throughput sequencing technology, such as next generation sequencing, has led to the discovery of a large number of ncRNAs, of which long ncRNAs are the largest, attracting great attention in the past decade. Integrated analysis of RNA-seq data revealed that 68% of human transcripts are lncRNAs with an estimated number of 55,000 to 60,000 [11]. LncRNAs are generally defined as transcripts longer than 200 nucleotides that do not have protein-coding potential; this relatively arbitrary cutoff distinguishes small ncRNAs from lncRNAs. Furthermore, lncRNAs are more similar to mRNAs than to other ncRNAs. First, the genome loci of lncRNA has a similar chromatin state to the mRNA genome loci, from which lncRNAs are transcribed by RNA polymerase II (Pol II) [12]. In addition, like mRNAs, lncRNAs are often polyadenylated, 5′-capped and spliced [13]. Therefore, analysis of whether open reading frames have protein-coding potential is an important means by which to distinguish lncRNAs from mRNAs. Relatively speaking, lncRNAs have lower expression levels, shorter transcripts, poorer sequence conservation and more nuclear enrichment than mRNAs [14].

There is no standard system for the identification and classification of lncRNA and their functions. This is likely due to the sheer number of lncRNAs, as well as their complex structures. In the past few years, reviews have attempted to classify lncRNAs by various molecular functions, including: (1) lncRNAs are divided into cis-acting lncRNAs and trans-acting lncRNAs by their genomic locus [15]. (2) lncRNAs are distinguished by the biological processes in which lncRNAs are involved [16, 17]. (3) Cancer-associated lncRNA are classified by their effects on cancer phenotype [18]. (4) HCC-associated lncRNAs are separated by their related signaling pathways [19]. In addition, lncRNAs can function via direct interaction with DNA, RNA and protein, making them versatile within biological processes. However, although lncRNAs have been extensively studied in recent years, the role of lncRNAs in the initiation and development of HCC remains to be characterized. Just as with lncRNAs in other fields, the identification and classification of these mysterious molecules in HCC are extremely challenging, ambiguous and full of exceptions. This review aims to classify the molecular mechanisms of HCC-associated lncRNAs comprehensively and clearly by combining lncRNA modes of molecular interaction with their involved biological processes (Additional file 1: Table S1).

## Classification of modes of molecular interaction of HCC-associated lncRNAs

Despite the diverse functions of lncRNAs [15], previous studies have shown that the primary way in which HCC-associated lncRNAs exert their biological roles is via interactions with DNA, RNA and proteins. To facilitate the understanding and categorization of these molecular modes of interaction, rare molecular interaction modes that have been elucidated under other physiological and pathological conditions would be excluded in this paper. The three molecular interactions described here represent the lncRNA molecular mechanisms that have been validated in HCC cell lines or tissues. (1) Sequester: The interactions of lncRNAs with DNA, RNA or proteins cause these molecules to be isolated from the original interacting molecules, thereby preventing the original interaction. (2) Scaffold: LncRNAs interact with various molecules to create linkages for these molecules and facilitate interaction between these molecules. (3) Guide (can be considered as a special case of scaffold): LncRNAs bind to transcription factors or chromatin-modifying complexes, directing these molecules to specific genomic sites to promote or inhibit the transcription of related genes (Additional file 1: Table S1).

## HCC-associated lncRNAs participate in various biological processes

The physiological and pathological effects of lncRNAs are achieved primarily through gene expression regulation. Transcription and translation are key phases of these biological processes [15, 16]. In HCC, lncRNAs are involved specifically in epigenetic regulation, transcription factor regulation, post-transcriptional regulation, and protein degradation. In addition, some lncRNAs affect protein modification and protein complex modulation in HCC; however, this classification is often rather subjective, as HCC-associated lncRNAs always regulate protein degradation by affecting protein ubiquitination, which is a type of protein modification [20–25]. Conversely, some protein modifications of transcription factors are regulated by HCC-associated lncRNAs [26, 27]. Yan et al. suggested that protein complex assembly affected by lncRNAs can influence protein function, thereby regulating the corresponding signaling pathway [28]. Thus, the molecular interaction modes of HCC-associated lncRNAs and the regulation of biological processes are diverse, and many details remain unclear. In this review, we propose a comprehensive, yet simple, method of combining modes of molecular interactions and biological processes to analyze the mechanisms by which lncRNAs exert their effects on HCC risk (Additional file 1: Table S1).

## HCC-associated lncRNAs in epigenetic regulation

A suitable chromatin state is crucial for gene expression. The relationship between chromatin state and nucleosome histone modification has been studied extensively. As an epigenetic repressor, polycomb repressive complex 2 (PRC2) can inhibit the transcription of various genes, and accelerate the development of HCC, through histone H3 lysine 27 (H3K27) trimethylation. However, as a trans-acting regulator, the mechanism by which PRC2 interacts with its target genes remains to be characterized [29].

**Fig. 1:**
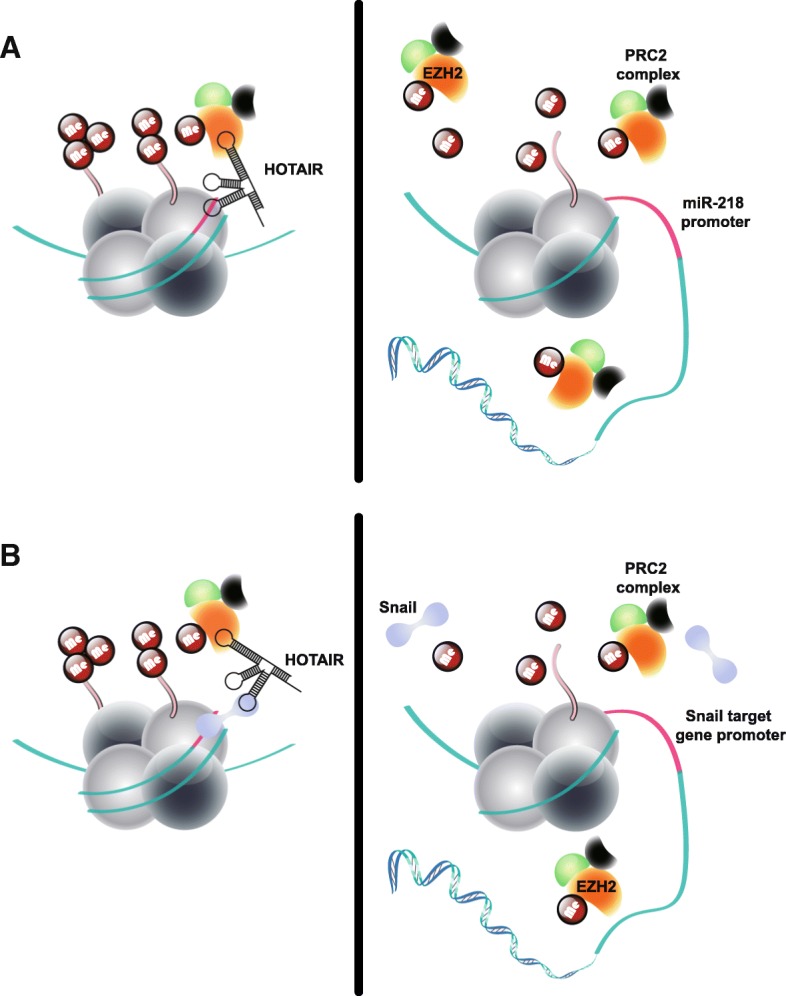
HCC-associated lncRNAs in histone modification. **a** HCC-associated lncRNAs recruit histone modifiers to regulate gene expression. Left panel: HOTAIR suppresses miR-218 expression by recruiting PRC2 to miR-218 promoter, which induces chromatin remodeling and H3K27 trimethylation. Right panel: decreased HORAIR inhibits PRC2-mediated miR-218 transcription suppression. **b** HCC-associated lncRNAs act as scaffold to bridge histone modifiers to regulate gene expression. Left panel: HOTAIR bridges PRC2 complex and Snail, which leads to H3K27 trimethylation in promoter region of Snail target genes. Right panel: decreased HORAIR inhibits PRC2-mediated transcription suppression of Snail target genes

In recent years, studies have indicated that 20% of lncRNAs can bind to PRC2 [30]. PRC2-associated lncRNAs have also been confirmed in HCC, among which the most recognized lncRNA may be HOX transcript antisense RNA (HOTAIR) [30, 31]. By directly interacting with the core subunit component enhancer of zeste homolog 2 (EZH2) of PRC2, HOTAIR can recruit PRC2 to the promoter region of miR-218, resulting in reduced expression of miR-218 (Fig. 1a). MiR-218 and Bmi-1 mRNA have a perfect seed pairing, which inhibits the activity of the P14ARF and P16Ink4a signaling pathway by reducing the translation of Bmi-1 [32]. According to the competing endogenous RNA (ceRNA) hypothesis, the microRNA binding sites on lncRNA or mRNA can titrate microRNAs (miRNAs) and regulate microRNA availability. Since it is easy to predict computationally, the lncRNA-miRNA-mRNA regulation axis is more likely to be interpreted by the ceRNA hypothesis [33]. Therefore, the mechanism by which lncRNAs inhibit miRNAs epigenetically is relatively inspiring. The molecular mechanism proposed by this study is novel and instructive on how to unravel the correlation between lncRNA and miRNA expression. Additionally, various HCC-associated lncRNAs have been shown to directly interact with EZH2 and inhibit the expression of different genes through a similar histone modification mechanism [34–39]. However, several studies have suggested the interaction between PRC2 and lncRNAs may also be promiscuous [40, 41] . Besides, HOTAIR has been reported to widely regulate the genome occupancy of PRC2, thus making it inappropriate to interpret this phenomenon simply in terms of DNA-RNA sequence complementarity [31], so this recruitment of PRC2 is likely to involve other molecules and mechanisms.

In fact, a previous study has proposed that the Snail protein can directly recruit PRC2 to epithelial targets [42]. During epithelial to mesenchymal (EMT) in HCC, HOTAIR acts as a scaffold to bridge PRC2 and Snail, which suppresses expression of HNF4a (hepatocyte nuclear factor 4, alpha), HNF1a (HNF1 homeobox A) and E-cadherin by Snail-dependent manner (Fig. 1b). The promoter regions of these genes contain E-box, a consensus Snail-binding sequence. When Snail is knocked down, it can significantly impair the HOTAIR-repressive activity on transcription of these Snail target genes [42].

HOTAIR has long been considered a risk factor for HCC [43, 44]. Interestingly, though, Zhang et al. found that the expressions of a human cancer stem cell marker, epithelial cell adhesion molecule (EpCAM), and pluripotent genes were increased by knocking down HOTAIR through siRNA transfection, thus making HOTAIR do not function as an oncogene [20]. Further, this research has shown that RNA Helicase DEAD Box Protein 5 (DDX5) is involved in this biologic process. Decreased levels of DDX5 suggests a poor prognosis for HCC patients. DDX5 can bind to HOTAIR and subunit suppressor of zeste 12 homolog (SUZ12), a PRC2 subunit, displacing an E3 ligase, Mex-3 RNA-binding family member B (Mex3b), from HOTAIR to inhibit Mex-3b-mediated SUZ12 degradation (Fig. 5b). DDX5 is thereby involved in the transcription inhibition of EpCAM and other pluripotency genes via the HOTAIR-PRC2 complex. Hepatitis B virus (HBV) infection downregulates DDX5 expression by the HBx protein, resulting in increased expression of the above-mentioned pluripotent genes. Therefore, HOTAIR regulates transcription inhibition and protein degradation simultaneously. Finally, two risk factors of HCC, HOTAIR and HBV infection, jointly promote the development of HCC [20]. Hence, some histone modifications in chromatin-modifying complexes in HCC are not only dependent on lncRNA, and the true mechanisms remain to be elucidated.

**Fig. 2:**
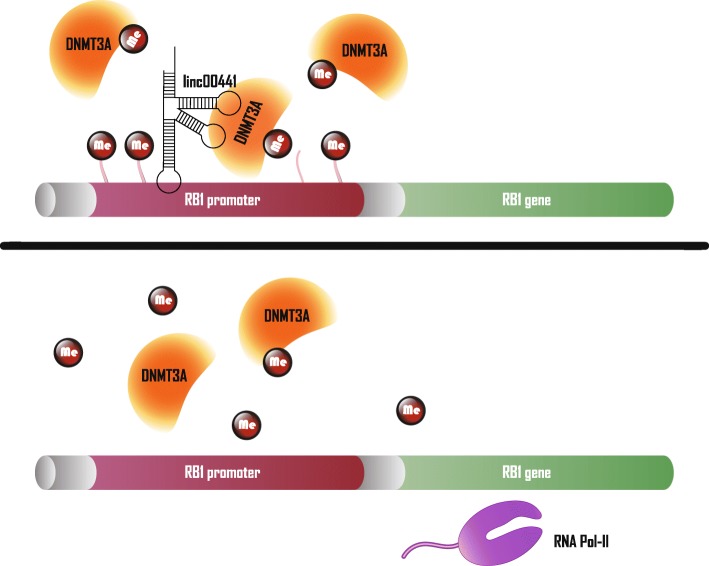
HCC-associated lncRNAs in DNA methylation modification. Upper panel: linc00441 suppresses RB1 expression by recruiting DNMT3A to RB1 promoter, which induces DNA methylation of RB1 promoter. Lower panel: decreased linc00441 inhibits DNMT3A-mediated RB1 transcription suppression

In addition to HOTAIR, some HCC-associated, lncRNA-mediated epigenetic regulations also demonstrate their complexity. For instance, lncRNA Gradually Increased During Hepatocarcinogenesis (GIHCG) not only regulates the transcription of miR-200a/b/429 via PRC2-mediated histone H3 lysine 27 trimethylation, but also methylates the histone promoter regions of these genes via DNA methyltransferase 1 (DNMT1). Thus, both regulatory mechanisms synergistically inhibit the expression of these genes [45]. Moreover, in addition to sharing a bidirectional promoter with retinoblastoma gene 1 (RB1), linc00441 also induces methylation of RB1 in the promoter region by recruiting DNA methyltransferase 3 (DNMT3), which reduces RB1 transcription (Fig. 2) [46].

In addition to regulating gene transcription by histone methylation and DNA methylation, HCC-associated lncRNAs, including H19 and GPC3 antisense RNA 1 (GPC3-AS1), also alter histone acetylation to influence HCC progression [47, 48]. Notably, lncTCF7 promotes liver CSC self-renewal by guiding the SWItch/Sucrose Non-Fermentable (SWI/SNF) complex to initiate transcription factor 7.

(TCF7) expression [49]. The SWI/SNF complex uses energy produced by ATP hydrolysis to directly mobilize nucleosomes and remodel chromatin, making it a distinctive epigenetic regulator [50]. Coupled with the above-described histone methylation, acetylation and DNA methylation, HCC-associated lncRNAs exhibit diverse capabilities in epigenetic regulation (Figs. 1 and 2). And the molecular interaction modes mentioned in this section are mainly guide and scaffold, further research in this field may help to characterize more mechanisms of lncRNA-based epigenetic regulation in HCC.

## HCC-associated lncRNAs in transcription factor regulation

**Fig. 3:**
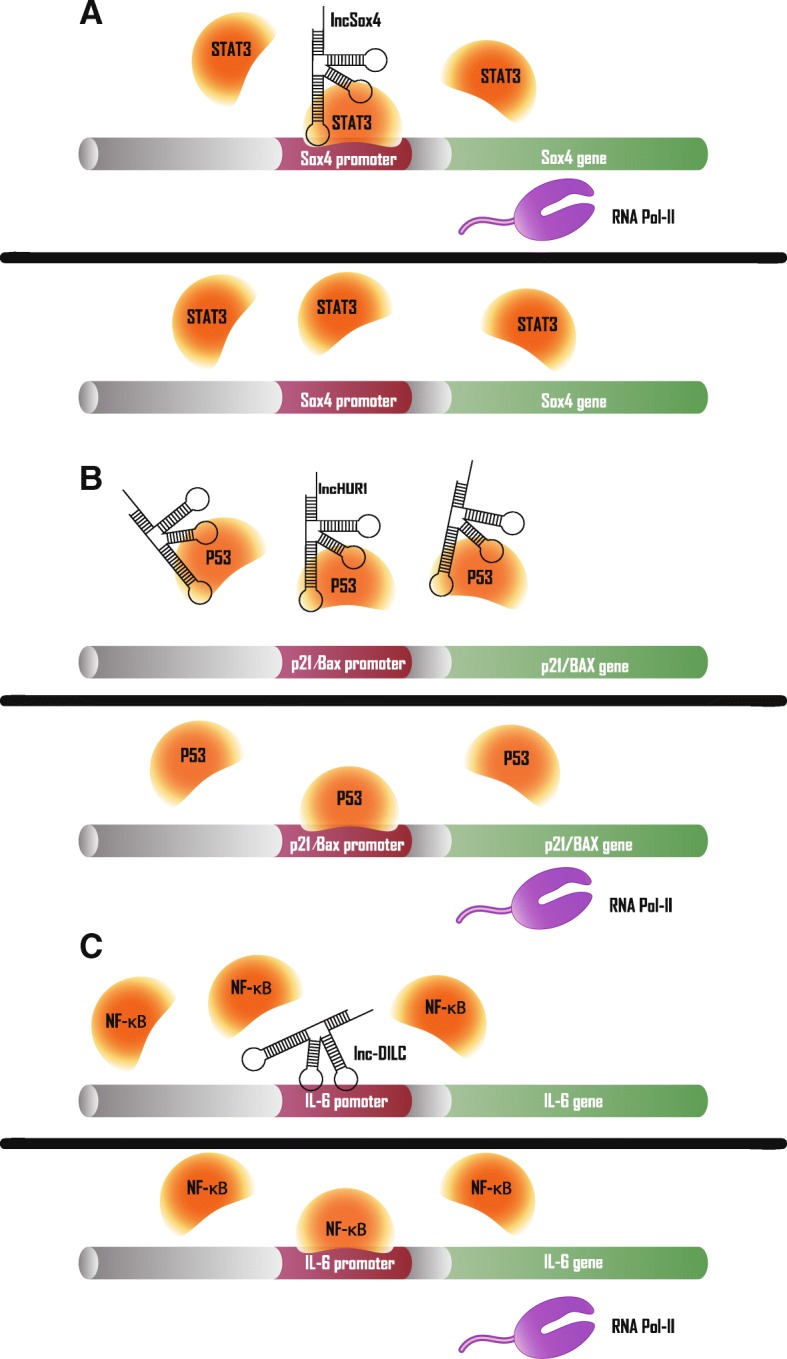
HCC-associated lncRNAs in transcription factors regulation. **a** Upper panel: lncSox4 recruits STAT3 to Sox4 promoter region to activate Sox4 transcription. Lower panel: decreased lncSox4 impairs STAT3-mediated Sox4 expression (**b**) Upper panel: lncHUR1 inhibits transcription of p21 and BAX by sequestering p53. Lower panel: decreased lncHUR1 releases p53 to bind to promoters of p21 and BAX, which activates transcription of them. **c** Upper panel: lnc-DILC inhibits NF-κB-mediated IL-6 transcription by blocking IL-6 promoter. Lower panel:NF-κB can bind to IL-6 promoter and promotes IL-6 transcription when lnc-DILC is decreased

In the nucleus, in addition to epigenetic regulation, lncRNAs can directly affect transcription factor function. Among them, lncSox4 (also known as cancer susceptibility 15, CASC15) is a nucleus-enriched lncRNA that is highly expressed in liver cancer and liver tumor-initiating cells (TIC). Mechanism investigation found that lncSox4 binds the sex determining region Y-box 4 (Sox4) promoter and recruits signal transducer and activator of transcription 3 (STAT3) to promote Sox4 expression, which is required for liver TIC self-renewal (Fig. 3a) [51]. Another HCC-associated lncRNA that regulates transcription factor function is lncWDR26; however, the result of this interaction is the transcriptional repression of WD repeat domain 26 (WDR26). As a tumor suppressor, SIX homeobox 3 (SIX3) can inhibit the expression of some metastasis and proliferation-related genes [52]. As a down-regulated lncRNA in HCC, the lncWDR26 recruits SIX3 to WDR26 promoter regions and represses WDR26 transcription [53]. In general, HCC-associated lncRNAs can directly guide transcription factors to their specific binding sites, leading to transcriptional activation or inhibition of related genes (Fig. 3a).

LncRNAs can also prevent transcription factors binding to their target promoters by interaction with them. For example, by comparing the transcriptome of HepG2 cells and HBV transgenic HepG2-4D14 cells, it was found that lnc-HUR1 transcription was enhanced by HBV-encoded HBx. Mechanistically, p53 is detained by lnc-HUR1, resulting in reduced promoter occupancy of the target gene, such as p21 and Bax (BAX). In conclusion, lnc-HUR1 can inhibit p53-promoted transcription of p21 and BCL2-associated X protein (Fig. 3b) [54]. Conversely, HCC-associated lncRNAs can also sequester transcription factors to allow some target gene release from transcription inhibition. For example, a cis-positive feedback loop exists for the tumor suppressor molecules lncRNA-NEF and forkhead box A2 (FOXA2). LncRNA-NEF is mainly distributed in the cytoplasm, while β-catenin binds the FOXA2 promoter region and inhibits FOXA2 transcription in the nucleus. The direct interaction of lncRNA-NEF with β-catenin increases the cytoplasmic abundance of β-catenin. At the same time, Glycogen synthase kinase 3 beta (GSK3β) also binds to, and increases the inhibitory phosphorylation of, β-catenin. Ultimately, lncRNA-NEF reduces nuclear levels of β-catenin, thereby increasing expression of FOXA2, which further promotes its transcription by binding lncRNA-NEF promoter [55].

Additionally, lncRNAs have been discovered that regulate gene expression by binding directly to the DNA element; that is, without interacting with transcription factors. A typical example of this in HCC is a lncRNA termed lncRNA downregulated in liver cancer stem cells (lnc-DILC). In contrast to lncSox4, down-regulated lnc-DILC in HCC inhibits STAT3 levels and suppresses liver cancer stem cell expansion. Using Basic Local Alignment Search Tool, Wang et al. identified a putative lnc-DILC complementary binding locus in the IL-6 promoter and verified that lnc-DILC inhibits NF-κB-mediated IL-6 transcription (Fig. 3c). Ultimately, lnc-DILC abolishes IL-6/JAK2/STAT3 autocrine signaling, and down-regulated lnc-DILC indicates poorer HCC prognosis [56]. Also, lncCAMTA1 plays a role in liver CSC expansion through similar mechanisms. As a transcript with higher expression levels in HCC and liver CSCs, lncCAMTA1 binds the calmodulin binding transcription activator 1 (CAMTA1) promoter and induces a suppressive chromatin structure, which leads to decreased CAMTA1 transcription. Subsequently reduced expression of CAMTA1 inhibits HCC cell proliferation and liver CSC-like properties [57]. Finally, TNF-α and IL-6 can stimulate expression of LINC000607, which represses NF-κB p65 transcription by binding to the NF-κB p65 promoter region, eventually causing apoptosis due to elevated p53 expression [58]. In brief, HCC-associated lncRNAs can reduce transcription factors-DNA complexes by sequestering one of them, thereby inhibiting the downstream effects (Fig. 3b, c).

## HCC-associated lncRNAs in mRNA post-transcriptional regulation

**Fig. 4:**
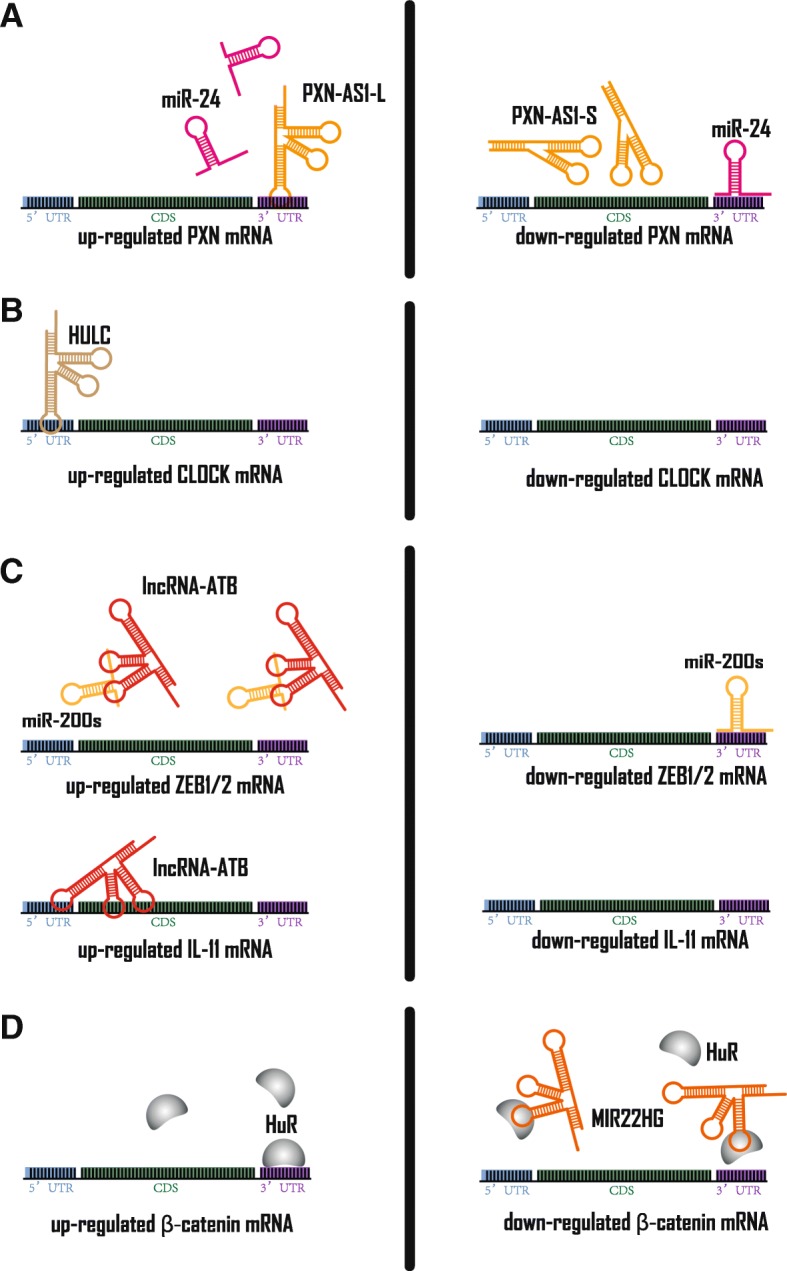
HCC-associated lncRNAs in mRNA post-transcriptional regulation. **a** Left panel: PXN-AS1-L can sequester miR-24 with its exon4, thereby increasing PXN mRNA stability. Right panel: PXN-AS1-S does not consist of exon4, which releases miR-24 to bind to PXN mRNA 3’UTR. **b** Left panel: HULC can increase CLOCK mRNA stability by binding to its 5’UTR. Right panel: decreased HULC expression impairs CLOCK expression. **c** Left panel: lncRNA-ATB can increase ZEB1/2 mRNA stability by sequestering miR-200 s. Also, lncRNA-ATB can increase IL-11 mRNA stability by binding with it. Right panel: decreased lncRNA-ATB expression impairs ZEB1/2 and IL-11 expression. **d** Left panel: HuR can stabilize β-catenin by binding to its 3’UTR. Right panel: MIR22HG can down-regulate β-catenin mRNA by sequestering β-catenin mRNA

Transcripts originating from various protein-coding genes in the nucleus require a series of post-transcriptional steps to generate mature RNAs before translation, including: 5′-end capping, alternative splicing and 3′-end cleavage/polyadenylation. Then, mRNAs are transported to cytoplasm by RNA-binding proteins and mRNA export receptors. Once in the cytoplasm, mRNAs are translated into proteins or targeted for decay [59]. HCC-associated lncRNAs regulate mature mRNA expression by directly binding to complementary sequences on target mRNA or miRNAs, thereby decreasing protein expression levels. Similarly, miRNAs can bind to mRNA 3′- Untranslated Region (UTR) and assemble into miRNA-induced silencing complexes with Argonaute family proteins to silence gene expression [60]. In general, the more complementary the seed region and mRNA target sequence, the stronger the corresponding protein expression changes [61]. LncRNAs that contain the same miRNA response elements as mRNAs can promote mRNA translation by ceRNA, also known as “miRNA sponges” [33]. In recent years, as bioinformatics tools have advanced, knowledge of the sequence complementarity between lncRNA and mRNA is more accessible. Thus, ceRNA has become a more pervasive molecular mechanism of HCC-associated lncRNAs. The most representative example of this is lncRNA-PXN-AS1. Muscleblind like splicing regulator 3 (MBNL3) acts as an oncofetal splicing factor to increase the stability of paxillin (PXN) mRNA by alternative splicing of PXN antisense transcript 1 (PXN-AS1). Specifically, overexpression of MBNL3 stimulates exon 4 inclusion of PXN-AS1, which raises PXN-AS1-L levels and lowers PXN-AS1-S levels. Compared to PXN-AS1-S, PXN-AS1-L’s unique exon 4 binds to the 3′-UTR region of PXN mRNA, which blocks miR-24 binding to this region and reduces degradation of PXN mRNA (Fig. 4a) [62]. Similarly, DANCR protects β-catenin from miR-214- or miR-320a-mediated degradation by binding to its mRNA 3′-UTR [63]. Interestingly, portal vein tumor thrombus-associated lncRNA ICAM-1–Related Noncoding RNA (ICR) and Intercellular Adhesion Molecule 1 (ICAM-1) mRNA originated from partially overlapping locations in different strands of chromosome 19, resulting in a complementary interaction between their respective, approximately 800 bp, regions at the 3′ end. These two transcripts are positively correlated in HCC, and ICR enhances the ICAM-1 mRNA stability by forming an RNA duplex with it, which may be due to blocking the binding site of certain miRNAs at the 3′-UTR [64].

In addition, some HCC-associated lncRNA-mRNA binding sites do not overlap with miRNA putative binding sites, but still affect the stability of the corresponding mRNAs. Hepatocellular Carcinoma Up-regulated Long Non-coding RNA (HULC) and Clock circadian regulator (CLOCK) mRNA 5′-UTR have a complementary base paring region, and the results of luciferase reporter gene assays suggest that HULC can improve the stability of CLOCK mRNA (Fig. 4b). Thereby, HULC disturbs circadian rhythm of HCC and accelerates hepatocarcinogenesis [65]. In contrast, lncARSR has been reported to promote the degradation of Phosphatase and tensin homolog (PTEN) mRNA by lncRNA-mRNA interaction in HCC, though the specific mechanism needs further characterization [66].

In addition to the above mechanism, to stabilize IL-11 mRNA and subsequently activate IL-11/STAT3 signaling, lncRNA-ATB can also indirectly improve mRNA stability and protein levels through the “miRNA sponge” model. Based on the TargetScan prediction algorithm, lncRNA-ATB was found to have three miR-200 s target binding sites in a short span. And it was confirmed by quantitative real-time PCR that lncRNA-ATB and miR-200 s have approximately 100 and 200 copies per cell in SMMC-7721 cells, respectively. These conditions meet ceRNA mechanism criteria. Finally, it was verified by luciferase reporter gene assays and MS2-RNA immunoprecipitation that lncRNA-ATB sequesters miR-200 s, thereby increasing the expression level of zinc finger E-box binding homeobox 1/2 (ZEB1/2) to induce epithelial-mesenchymal transition (Fig. 4c) [67]. This pattern is the most widely studied molecular mechanism in HCC-associated lncRNAs, revealing the ubiquitous lncRNA-miRNA-mRNA axis in HCC (Additional file 1: Table S1).

The above-mentioned mRNA post-transcriptional regulation has an obvious cellular compartment context. A study of two lncRNAs, lncRNA-UFC1 and MIR22 host gene (MIR22HG), supports the significant impact of subcellular location of some molecules on certain biological processes, especially mRNA post-transcriptional regulation. For instance, HuR (also known as ELAV like RNA binding protein 1) is a mRNA stabilizing protein for which its deregulated nucleus: cytoplasm ratio leads to tumor initiation and progression [68]. Interestingly, the interaction of HuR and Adenylate-uridylate-rich elements in the 3′ UTR can enhance mRNA stability. β-catenin mRNA is one such example [69]. LncRNA-UFC1 acts as an HCC promoter by increasing cytoplasmic HuR levels, which results in more stable β-catenin mRNA [70]. Conversely, MIR22HG, a down-regulated lncRNA in HCC, competitively binds HuR with β-catenin and increases the nuclear fraction of HuR (Fig. 4d). That is to say, MIR22HG reduces β-catenin level by altering subcellular location of its mRNA stabilizing protein HUR [71]. Therefore, in addition to epigenetic modulation and transcription factor regulation, mRNA post-transcriptional regulation is another subcellular, location-dependent lncRNA mode of mechanism of action in HCC.

## HCC-associated lncRNAs in protein post-translational regulation and protein complex modulation

HCC-associated lncRNAs regulate protein post-translationally via several molecular patterns. In protein degradation, lncRNAs promote or inhibit ubiquitination of proteins, which subsequently affects ubiquitin–proteasome-mediated protein degradation. LncRNAs can also modify proteins, represented by protein phosphorylation to have varying effects on their functions. Moreover, proteins often bind to each other to form protein complexes, and some HCC-associated lncRNAs transform the composition of subunits in various complexes. In these ways, HCC-associated lncRNAs affect protein post-translationallyand regulate the assembly of multiprotein complex .

### Protein degradation

There are multiple pathways for protein degradation in humans, which are generally classified into (1) degradation of dietary proteins, (2) degradation of extracellular proteins, and (3) degradation of intracellular proteins. Among them, the ubiquitin proteasome system (UPS) is a highly-specific cellular mode of protein degradation that plays a key role in maintaining protein quality and controlling cellular processes [72]. HCC-associated lncRNAs generally affect protein degradation through UPS, thereby regulating a series of signaling pathways.

**Fig. 5:**
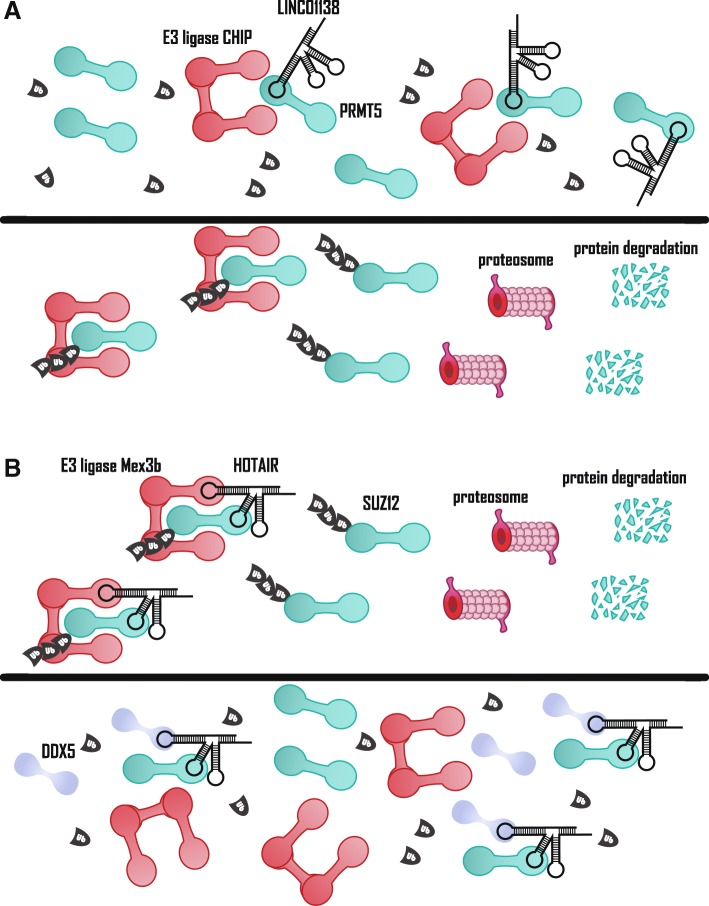
HCC-associated lncRNAs in protein degradation. **a** Upper panel: LINC01138 blocks E3 ligase CHIP-mediated ubiquitination of PRMT5 by sequestering PRMT5. Lower panel: decreased LINC01138 leads to release of PRMT5, which leads to E3 ligase CHIP-mediated ubiquitination of PRMT5 and its degradation. **b** Upper panel: HOTAIR acts as scaffold to bridge E3 ligase Mex3b and SUZ12, a core subunit of PRC2 complex, which leads to ubiquitination and degradation of SUZ12. Lower panel: DDX5 inhibits E3 ligase Mex3b-mediated SUZ12 protein degradation by displacing the Mex3b from HOTAIR

26S proteasome recognizes ubiquitin-conjugated proteins and degrades them into small peptides. The process of ubiquitination requires E1 (ubiquitin-activating enzyme), E2 (ubiquitin-conjugating enzyme), and a substrate-specific E3 (ubiquitin-protein ligase) [72]. The interaction between E3 ligase and its target protein can be blocked by lncRNAs through sequestration. The direct interaction of E3 ligase Carboxy-Terminus of Hsc70 Interacting Protein (CHIP) with arginine methyltransferase 5 (PRMT5) prompted proteasomal degradation of PRMT5. LINC01138 was found to increase the protein level of PRMT5, but had no effect on the level of PRMT5 mRNA. Mechanistically, LINC01138 withholding PRMT5 results in significant inhibition of the association between CHIP and PRMT5 (Fig. 5a). Gene set enrichment analysis shows that LINC01138 and PRMT5 affect highly similar downstream signaling pathways, which may demonstrate that PRMT5 is a mediator of LINC01138’s oncogenic role [73]. Furthermore, lnc-epidermal growth factor receptor (EGFR) similarly enhances EGFR stability, thereby increasing Treg cell differentiation and promoting the immunosuppressive state of HCC. More specifically, the domain (1001–1051 amino acids) of EGFR is exposed in the cytoplasm and is responsible for the physical interaction with lnc-EGFR, where Tyr1045 is the docking site of E3 ligase casitas B-lineage lymphoma (c-CBL). Collectively, lnc-EGFR inhibits the ubiquitination and degradation of subsequent EGFR by blocking the binding site of E3 ligase c-CBL, thereby promoting HCC progression [74].

In addition to sequestering, HCC-associated lncRNAs can also act as a scaffold to bridge the interaction between E3 ligase and its target protein to promote protein degradation. The DDX5 mentioned above directs the HOTAIR-PRC2 complex to epigenetically inhibit the transcription of specific genes. The human cancer stem cell marker EpCAM and pluripotency genes Nanog homebox, Oct4 (also known as POU class 5 homeobox 1) and Sox2 do not match HOTAIR’s role as an oncogenic factor [31]. Zhang et al. found that in the presence of HBx, the E3 ligase Mex3b had more affinity for the PRC2 subunit SUZ12 and HOTAIR, whereas the interaction between DDX5 and these two molecules was reduced. As a replacement, Mex3b ubiquitinates SUZ12 and induces its degradation (Fig. 5b). HOTAIR acts as a molecular scaffold in both epigenetic regulation and ubiquitination, selectively affects gene expression, and drives HBV-induced liver tumors with HBx [20]. Conversely, HCC-associated lncRNAs can also bind deubiquitinase and target proteins to inhibit proteolysis. For example, one of the pathways that oncogenic lncRNA LNC473 regulates links survivin and deubiquitinase ubiquitin specific peptidase 9 (USP9X) to suppress the ubiquitination level of survivin and increase survivin expression [75].

**Fig. 6:**
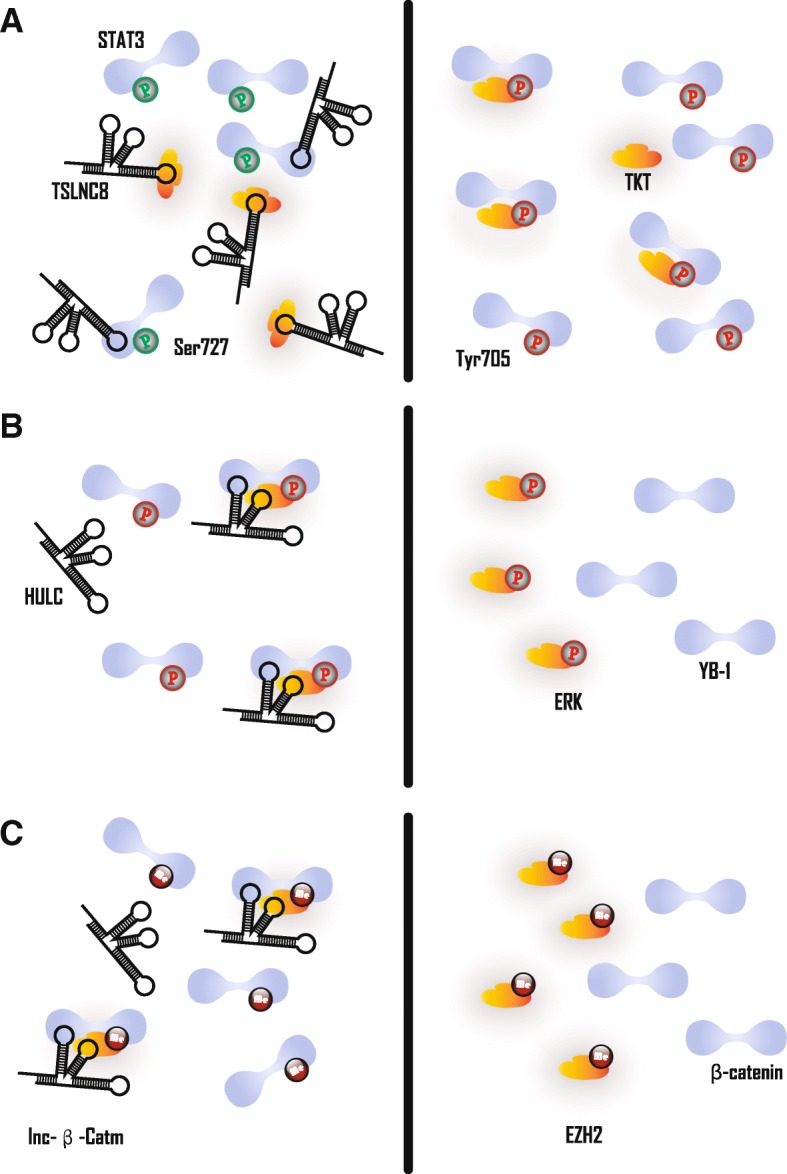
HCC-associated lncRNAs in protein modification. **a** Left panel: TSLNC8 disrupts TKT-mediated STAT phosphorylation by sequestering STAT3 or TKT, which induces Ser727 phosphorylation and Tyr705 dephosphorylation in STAT3. Right panel: decreased TSLNC8 promotes the interaction between STAT3 and TKT, which induces TKT-mediated Ser727 dephosphorylation and Tyr705 phosphorylation in STAT3. **b** Left panel: HULC promotes ERK mediated YB-1 phosphorylation by acting as scaffold to bridge ERK and YB-1. Right panel: decreased HULC impairs ERK mediated YB-1 phosphorylation. **c** Left panel: lnc-β-Catm promotes ERK mediated β-catenin methylation by acting as scaffold to bridge EZH2 and β-catenin. Right panel: decreased lnc-β-Catm impairs EZH2 mediated β-catenin methylation

However, the effect of lncRNA on UPS-mediated protein degradation is not limited to the ubiquitination of target proteins. The lnc-b-Catm is a lncRNA that mediates protein methylation, which is identified by comparing CD13+ CD133+ cells with CD13–CD133– cells. Lnc-b-Catm acts as a scaffold to enhance the interaction between EZH2 and β-catenin, thus causing EZH2 to methylate β-catenin at K49 (Fig. 6c). This methylation then inhibits β-catenin’s phosphorylation and subsequent ubiquitination, which inhibits degradation of β-catenin [76]. Furthermore, some HCC-associated lncRNAs bind proteins to increase their stability and expression levels, but understanding how the ubiquitination levels of these molecules requires further investigation [24, 25, 77].

### Protein phosphorylation

Similar to ubiquitination, HCC-associated lncRNAs also regulate protein phosphorylation primarily through two molecular modes of interaction: scaffolding and sequestering. Tumor suppressorlong noncoding RNA on chromosome 8p12 (TSLNC8) regulates phosphorylation of T705 and S727 on STAT3 by a relatively specific manner of sequestration. In short, lncRNAs interact with one of the two molecules, thereby inhibiting the interaction between them. Intriguingly, the right arm of TSLNC8 can be combined with transketolase (TKT) or STAT3 to allow the two molecules to compete with each other. That is, TSLNC8, TKT and STAT3 can combine with each other, but the presence of TSLNC8 reduces the interaction between TKT and STAT3. Eventually, overexpression of TLSNC8 results in a decrease in STAT3 Y705 phosphorylation and an increase in S727 phosphorylation, which can significantly attenuate the oncogenic ability of STAT3 (Fig. 6a) [26]. Unlike TSLNC8, HULC can simultaneously bind Y-box binding protein 1 (YB-1) and extracellular signal-regulated kinase (ERK) to promote phosphorylation of YB-1 by ERK (Fig. 6b). Phosphorylation of YB-1 results in the release of some mRNAs, which ultimately accelerates the translation of these mRNAs. The resulting increase in cyclin D1 and cyclin E1 promotes G1/S transition. This represents another mechanism by which HCC can develop due to HULC action [27]. HCC associated long non-coding RNA (HANR) appears to detain the GSK3B-interacting protein (GSKIP), which hinders GSKIP-mediated GSK-3β phosphorylation. GSK-3β promotes the phosphorylation of β-catenin and causes it to be hydrolyzed by the proteasome pathway. Therefore, HANR is also an HCC oncogenic factor that affects protein phosphorylation [78, 79].

Furthermore, Ding et al. proposed that HNF1A antisense RNA 1 (HNF1A-AS1) modulates the phosphatase activity of SH2-containing protein tyrosine phosphatase-1 (SHP-1) by binding with its C-terminal. Nevertheless, how such interaction enhances the enzymatic activity of SHP-1 remains to be characterized [80].

### Protein complex modulation

**Fig. 7:**
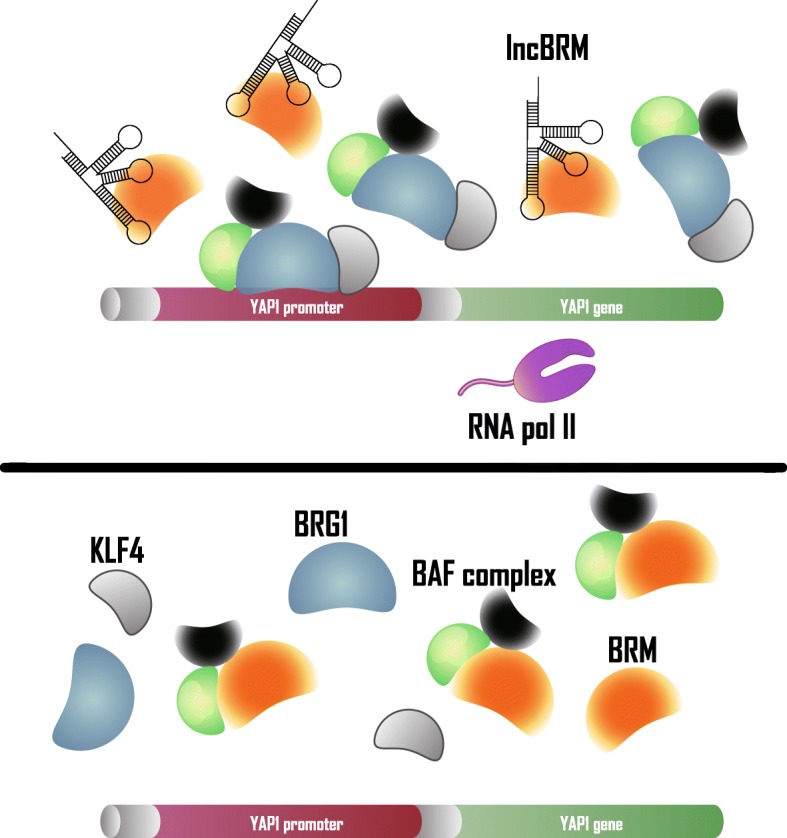
HCC-associated lncRNAs in protein complex modulation. Upper panel: lncBRM regulates assembly of BAF complex by sequestering BRM, which leads to BRG1-embedded BAF complex being directed by KLF4 and binding with YAP1 promoter to activate YAP1 transcription subsequently. Lower panel: decreased lncBRM induces release of BRM, which facilitates BRM/BRG1 switch in BAF complex and inhibits YAP1 transcription

The molecular mode of interaction of HCC-associated lncRNAs in protein complex modulation is the same as in the first two sections; that is, by sequestering and as a scaffold. First, lncBRM indirectly facilitates the assembly of a subunit into the BRG1-associated factor (BAF) complex by splitting another subunit, which is a type of subunit switch. LncBRM, another lncRNA derived by comparing CD13 + CD133+ cells and CD13-CD133-cells transcriptome, binds to Brahma (BRM), resulting in a decrease in BRM-embedded BAF complex. Correspondingly, the BRG1 act as a substitute for BRM to form the BRG1-embedded BAF complex. The difference between the two complexes is that BRG1 can bind to transcription factor Kruppel-like factor 4 (KLF4), while there is no interaction between BRM and KLF4. The BRG1-embedded BAF complex is directed by KLF4 to the Yes-associated protein 1 (YAP1) promoter, which facilitates YAP1 transcription (Fig. 7). Therefore, the increase in lncBRM ultimately promotes the LCSC property of cells by activating the BAF/YAP1 pathway [81].

Another example is lnc-Tim3-mediated HCC CD8 T lymphocyte exhaustion, which compromises HCC anti-tumor immunity. Briefly, Bat3 can simultaneously bind the C-terminal intracellular domain of Tim-3 (also known as hepatitis A virus cellular receptor 2) and Lck (also known as Src family tyrosine kinase). The formation of this complex activates T cell signaling (ZAP70/AP-1/NFAT1) and suppresses CD8 T cell exhaustion. However, lnc-Tim3 interferes with this process by binding the C-terminal intracellular domain of Tim-3, thereby releasing BAT3 from Tim3. BAT3 is then free to form a complex with p300 and recruit p300 into the nucleus, which exacerbates CD8 T cell exhaustion [82]. A similar phenomenon also occurs in the Wnt/β-catenin signaling pathway regulated by Linc00210 in TIC. The regulation of the Wnt/β-catenin signaling pathway is dependent on the alternative assembling of proteins as subunits in various complexes [79]. Linc00210 binds to catenin beta interacting protein 1 (CTNNBIP1) and impairs the interaction between β-catenin and CTNNBIP1. CTNNBIP1 acts as a Wnt/β-catenin signaling pathway suppressor that can detain and prevent β-catenin from binding to TCF/LEF components. Without β-catenin, TCF/LEF components act as transcription repressors. β-catenin is an activator of TCF/LEF components to release the repressed gene. Through this series of consecutive steps, Linc00210 became an HCC-related oncogene by means of protein complex modulation [83]. In addition, as described above, GSK-3β can inhibit β-catenin by phosphorylation, and the interaction between GSK-3β and Annexin A2 is enhanced by scaffolding of lncRNA-MUF. Eventually, β-catenin escapes inhibition by GSK-3β, and is thus able to accumulate and translocate to nucleus [28].

Although different in biological processes, HCC-associated lncRNAs regulate protein degradation, protein modification, and protein complexes primarily by affecting interactions between other molecules. In short, HCC-associated lncRNAs affect these biological processes through two molecular interaction modes: sequester and scaffold. However, HCC-associated lncRNAs may not be just a molecular interaction partner, although this assumption requires stronger evidence to support.

## Conclusion and perspective

As the largest class of transcripts in humans, lncRNAs can affect almost any realm of cellular biology. In order to fully understand the cellular mechanisms underlying the development of diseases such as HCC, it is necessary to understand lncRNA function. To our knowledge, no comprehensive model yet exists for the classification of HCC-associated lncRNAs. There is a considerable contribution by lncRNAs to the “transcriptional noise” that impacts the central dogma of the “DNA-RNA-protein” axis. Therefore, this review focuses on transcription and translation, classifies lncRNAs according to biological processes, and further subdivides them by their most common modes of molecular interaction in HCC. This newly streamlined classification method provides a more approachable system by which to study and discuss lncRNAs in HCC. We must note, however, there are still exceptional HCC-associated lncRNAs that are not suitable for this classification method, such as MVIH and Dreh [84, 85]. Moreover, some lncRNAs have been characterized to regulate gene expression via other mechanisms under other physiological and pathological conditions. For instance, lncRNA termed functional intergenic repeating RNA element (Firre), that promotes cross-chromosomal interactions as a trans-acting platform in several cell lines [86], while Colorectal Cancer Associated Transcript 1-L facilitates long-range interactions between MYC promoter and its enhancers in cis in colorectal cancer [87]. And lncRNAs generated from Alu SINE elements can repress the transcription machinery directly by binding to RNA polymerase II (Pol II) during heat shock [88]. Given that lncRNAs comprise 68% of the human transcriptome, future research may reveal new categories or definitions of lncRNAs.

High-throughput sequencing technology, followed by functional studies, have helped to identify and elucidate the role of a large number of lncRNAs in HCC over the past decade. However, the vast majority of lncRNAs still need to be investigated. Given the worldwide impact on morbidity and mortality by HCC, it is important that future research focuses on lncRNAs. In addition, the mechanisms of aberrant gene expression caused by lncRNAs in HCC has been more explicitly studied (Additional file 1: Table S1). Interestingly, it has also been noted that prostate cancer-associated single nucleotide polymorphisms (SNPs) are mainly enriched in regulatory regions, some of which are located in lncRNAs and can affect the their functions [89]. A similar association may exist for HCC, and by integrating genome-wide association studies and transcriptome data, it may be possible to discover and elucidate the mechanisms of some lncRNA-related SNPs. This has been practiced in several studies of various cancers, including HCC [89–94]. In the near future, research on the role of SNP-mediated HCC-related lncRNAs should be rewarded [95, 96], as this progress may become the key to gaining a greater understanding of the development of HCC.

## Additional file

Additional file 1: **Table S1.** Summary of HCC-associated lncRNAs. (DOCX 171 kb)

## Abbreviations

BAF: BRG1-associated factor
BAX: BCL2-associated X protein
BRM: Brahma
CAMTA1: calmodulin binding transcription activator 1
c-CBL: casitas B-lineage lymphoma
ceRNA: competing endogenous RNA
CHIP: Carboxy-Terminus of Hsc70 Interacting Protein
CLOCK: Clock circadian regulator
CTNNBIP1: catenin beta interacting protein 1
DDX5: RNA Helicase DEAD Box Protein 5
DNMT1: DNA methyltransferase 1
DNMT3: DNA methyltransferase 3
EGFR: Epidermal growth factor receptor
EMT: Epithelial to mesenchymal
EpCAM: Epithelial cell adhesion molecule
ERK: Extracellular signal-regulated kinase
EZH2: Enhancer of zeste homolog 2
Firre: Functional intergenic repeating RNA element
FOXA2: Forkhead box A2
GIHCG: Gradually Increased During Hepatocarcinogenesis
GPC3-AS1: GPC3 antisense RNA 1
GSK3β: Glycogen synthase kinase 3 beta
GSKIP: GSK3B-interacting protein
HANR: HCC associated long non-coding RNA
HBV: Hepatitis B virus
HCC: Hepatocellular carcinoma
HNF1a: HNF1 homeobox A
HNF1A-AS1: HNF1A antisense RNA 1
HNF4a: Hepatocyte nuclear factor 4, alpha
HOTAIR: HOX transcript antisense RNA
HULC: Hepatocellular Carcinoma Up-regulated Long Non-coding RNA
ICAM-1: Intercellular Adhesion Molecule 1
ICR: ICAM-1–Related Noncoding RNA
KLF4: Kruppel-like factor 4
lnc-DILC: lncRNA downregulated in liver cancer stem cells
lncRNAs: long non-coding RNAs
MBNL3: Muscleblind like splicing regulator 3
Mex3b: Mex-3 RNA-binding family member B
MIR22HG: MIR22 host gene
miRNAs: microRNAs
Pol II: RNA polymerase II
PRC2: Polycomb repressive complex 2
PRMT5: Arginine methyltransferase 5
PTEN: Phosphatase and tensin homolog
PXN: Paxillin
PXN-AS1: PXN antisense transcript 1
RB1: Retinoblastoma gene 1
SHP-1: SH2-containing protein tyrosine phosphatase
SIX3: SIX homebox 3
SNPs: Single nucleotide polymorphisms
Sox4: Sex determining region Y-box 4
STAT3: Signal transducer and activator of transcription 3
SUZ12: Subunit suppressor of zeste 12 homolog
SWI/SNF: SWItch/Sucrose Non-Fermentable
TCF7: Transcription factor 7
TIC: Tumor-initiating cells
TKT: Transketolase
TSLNC8: Tumor suppressor long noncoding RNA on chromosome 8p12
UPS: Ubiquitin proteasome system
USP9X: Ubiquitin specific peptidase 9
UTR: Untranslated Region
WD: Repeat domain 26
YAP1: Yes-associated protein 1
YB-1: Y-box binding protein 1
ZEB1/2: Zinc finger E-box binding homeobox 1/2
