# Correction to: Molecular pattern of lncRNAs in hepatocellular carcinoma

**DOI:** 10.1186/s13046-019-1339-0

**Published:** 2019-08-14

**Authors:** Haoming Mai, Bin Zhou, Li Liu, Fu Yang, Carly Conran, Yuan Ji, Jinlin Hou, Deke Jiang

**Affiliations:** 1 State Key Laboratory of Organ Failure Research, Guangdong Key Laboratory of Viral Hepatitis Research, Institute of Liver Diseases Research of Guangdong Province, Department of Infectious Diseases and Hepatology Unit, Nanfang Hospital, Southern Medical University, GuangZhou, 510515 China; 2 Department of Medical Genetics, Second Military Medical University, Shanghai, 200433 China; 3 University of Illinois College of Medicine, Chicago, IL 60612 USA; 4 Department of Public Health Sciences, University of Chicago, Chicago, IL 60637 USA

**Correction to: J Exp Clin Cancer Res**

**https://jeccr.biomedcentral.com/articles/10.1186/s13046-019-1213-0**

In the original publication of this article [1], the author’ affiliations need to be revised, because the first and second affiliations should combine as the same affiliation.

The correct version is:

Haoming Mai^1^, Bin Zhou^1^, Li Liu^1^, Fu Yang^2^, Carly Conran^3^, Yuan Ji^4^, Jinlin Hou^1^ and Deke Jiang^1*^

^1^State Key Laboratory of Organ Failure Research, Guangdong Key Laboratory of Viral Hepatitis Research, Institute of Liver Diseases Research of Guangdong Province, Department of Infectious Diseases and Hepatology Unit, Nanfang Hospital, Southern Medical University, GuangZhou 510515, China

^2^Department of Medical Genetics, Second Military Medical University, Shanghai 200433, China

^3^University of Illinois College of Medicine, Chicago, IL 60612, USA

^4^Department of Public Health Sciences, University of Chicago, Chicago, IL 60637, USA

## References

[1] Mai (2019). J Exp Clin Cancer Res.

