# Supplementary material for: Molecular pattern of lncRNAs in hepatocellular carcinoma
Source: J Exp Clin Cancer Res. 2019 May 16;38:198. doi: 10.1186/s13046-019-1213-0 (PMC6524221; doi:10.1186/s13046-019-1213-0)
Supplement: Supplementary file 1 — Table S1. Summary of HCC-associated lncRNAs. (DOCX 171 kb) [file 13046_2019_1213_MOESM1_ESM.docx]

**Table S1. Summary of HCC-associated lncRNAs**

| **lncRNAs** | **Target molecule and its classification** | | **Biological process** | **Molecular mode of interaction** | **Subcellular location** | **Biological outcome** | **Aberrant expression and its cause** | |
| --- | --- | --- | --- | --- | --- | --- | --- | --- |
| ANRIL [1] | protein/DNA | KLF2 promoter and EZH2 | epigenetic regulation | guide | nucleus | ANRIL inhibits KLF2 transcription and recruiting PRC2 to the KLF2 promoter | up-regulation | SP1 promotes ANRIL transcription by binding its  promoter |
| GHET1 [2] | protein/DNA | KLF2 promoter and EZH2 | epigenetic regulation | guide | nucleus | GHET1 inhibits KLF2 transcription by recruiting PRC2 to KLF2 promoter | up-regulation |  |
| GIHCG [3] | protein/DNA | DNMT1 and miR-200a/b/429 promoter | epigenetic regulation | guide | nucleus | GIHCG inhibits miR-200b/a/429 transcription by recruiting DNMT1 to miR-200b/a/429 promoters | up-regulation |  |
| GIHCG [3] | protein/DNA | EZH2 and miR-200a/b/429 promoter | epigenetic regulation | guide | nucleus | GIHCG inhibits miR-200b/a/429 transcription by recruiting EZH2 to miR-200b/a/429 promoters | up-regulation |  |
| GPC3-AS1 [4] | protein/DNA | P300/CBP-associated factor | epigenetic regulation | guide | nucleus | GPC3-AS1 promotes GPC3 transcription by recruiting P300/CBP-associated factor to GPC3 gene body | up-regulation |  |
| H19 [5] | protein/DNA | hnRNP U and miR-218 promoter | epigenetic regulation | guide | nucleus and cytoplasm | H19 promotes miR-200 transcription by recruiting the hnRNP U/PCAF/RNA PolII complex to miR-200 promoter | up-regulation | Sox2 promotes H19 transcription by binding its  promoter[6] |
| HNF1A-AS1 [7] | protein/DNA | EZH2(PRC2) and NKD1/p21 promoter | epigenetic regulation | guide | nucleus and cytoplasm | HNF1A-AS1 inhibits NKD1 and p21 transcription by recruiting PRC2 to NKD1 and p21 promoter | up-regulation |  |
| HOTAIR [7] | protein/DNA | EZH2(PRC2) and miR-218 promoter | epigenetic regulation | guide | nucleus and cytoplasm | HOTAIR inhibits miR-218 transcription by recruiting PRC2 to miR-218 promoter | up-regulation |  |
| linc00441 [8] | protein/DNA | DNMT3A/RB1 promoter | epigenetic regulation | guide | nucleus | Linc00441 inhibits RB1 transcription by recruiting DNMT3A to RB1 promoter | up-regulation | TCF-4 promotes the transcription and H3K27Ac of Linc00441. |
| LncDQ [9] | protein/DNA | E-cadherin promoter and EZH2(PRC2) | epigenetic regulation | guide |  | LncDQ inhibits E-cadherin transcription by recruiting PRC2 to E-cadherin promoter | up-regulation |  |
| lncRNA-HEIH [10] | protein/DNA | EZH2(PRC2) and its target genes' promoter | epigenetic regulation | guide | nucleus and cytoplasm | lncRNA-HEIH inhibits PRC2 target genes transcription by recruiting PRC2 to their promoters | up-regulation |  |
| lncTCF7 [11] | Protein | SWI/SNF complex and TCF7 promoter | epigenetic regulation | guide | nucleus | LncTCF7 promotes TCF7 transcription by recruiting the SWI/SNF complex to TCF7 promoter | up-regulation | IL-6-STAT3 induces lncTCF7 transcription via STAT3 binding lncTCF7 promoter [12] |
| SOX21-AS1 [13] | protein/DNA | P21 promoter and EZH2(PRC2) | epigenetic regulation | guide |  | SOX21-AS1 inhibits P21 transcription by recruiting PRC2 to P21 promoter | up-regulation |  |
| SPRY4-IT1 [14] | protein/DNA | E-cadherin promoter and EZH2(PRC2) | epigenetic regulation | guide |  | SPRY4-IT1 inhibits E-cadherin transcription by recruiting PRC2 to E-cadherin promoter | up-regulation |  |
| TUG1 [15] | protein/DNA | KLF2 promoter and EZH2(PRC2) | epigenetic regulation | guide | nucleus | TUG1 inhibits KLF2 transcription by binding with PRC2 and recruiting it to the KLF2 promoter | up-regulation | SP1 promotes TUG1 transcription by binding TUG1 promoter |
| HOTAIR [16] | protein/DNA | Snail, EZH2(PRC2) and Sna·il target promoters | epigenetic regulation | scaffold | nucleus and cytoplasm | HOTAIR bridges the interaction of Snail and EZH2, while Snail directs the HOTAIR/Snail/EZH2 complex to its target genes promoter | up-regulation |  |
| lncRNA-LALR1 [17] | protein/DNA | CTCF and AXIN1 promoter | transcription factor regulation | guide | nucleus | lncRNA-LALR1 inhibits the transcription of Axin1 by recruiting CTCF to AXIN1 promoter | up-regulation |  |
| lncSox4 [18] | protein/DNA | STAT3 and Sox4 promoter | transcription factor regulation | guide | nucleus | lncSox4 promotes Sox4 transcription by recruiting STAT3 to Sox4 promoter | up-regulation |  |
| lncWDR [19] | protein/DNA | SIX3 and WDR26 promoter | transcription factor regulation | guide | nuclei | lncWDR26 inhibits WDR26 transcription by recruiting SIX3 to WDR26 promoter | down-regulation |  |
| LINC000607 [20] | DNA | NF-κB p65 promoter | transcription factor regulation | sequester | nucleus and cytoplasm | LINC000607 inhibits NF-κB p65 transcription by binding NF-κB p65 promoter | down-regulation | LINC000607 is up-regulated by TNF-α and IL-6 stimulation in MHCC97H liver cancer cells |
| lncCAMTA1 [21] | DNA | CAMTA1 promoter | transcription factor regulation | sequester | nucleus | lncCAMTA1 inhibits CAMTA1 transcription by binding CAMTA1 promoter | up-regulation |  |
| lnc-DILC [22] | DNA | IL-6 promoter | transcription factor regulation | sequester | nucleus | lnc-DILC inhibits STAT3 transcription by binding STAT3 promoter | down-regulation |  |
| lnc-HUR1 [23] | Protein | p53 | transcription factor regulation | sequester | nucleus | lnc-HUR1 inhibits p21 and Bax transcription activation by sequestering p53 | up-regulation | HBx can enhance the transcription activity of lnc-HUR1. |
| LncRNA-NEF [24] | Protein | β-catenin | transcription factor regulation | sequester | nucleus | LncRNA-NEF promotes FOXA2 transcription by sequestering β-catenin | down-regulation | FOXA2 promotes LncRNA-NEF by binding its promoter |
| WT1-AS [25] | DNA | Wilms’tumor 1(WT1) promoter | transcription factor regulation | sequester | nucleus | WT1-AS binds to TATA sequence of WT1 promoter to inhibit its transcription | down-regulation |  |
| CASC9[26] | protein | Heterogeneous nuclear ribonucleoprotein L(HNRNPL) | mRNA post-transcriptional regulation |  | Cytoplasm | the CASC9-HNRNPL complex regulate PI3K/AKT-signaling cascade by an unknown mechanism | up-regulation |  |
| lincRNA-UFC1 [27] | protein | HuR | mRNA post-transcriptional regulation | guide | cytoplasm | lincRNA-UFC1 interacts with HuR and increases its cytoplasmic abundance | up-regulation | miR-34a can bind to lincRNA-UFC1 and reduce its expression |
| lnc-UCID[28] | protein | DEAH box helicase 9 (DHX9) | mRNA post-transcriptional regulation | sequester | nucleus and cytoplasm | lnc-UCID promotes CDK6 translation by sequestering DHX9 | up-regulation |  |
| MIR22HG [29] | protein | HuR | mRNA post-transcriptional regulation | sequester | cytoplasm | MIR22HG interacts with HuR and increases its nuclear abundance | down-regulation |  |
| RP11-598D14.1[30] | protein | insulin-like growth factor 2 mRNA binding protein 1(IGF2BP1) | mRNA post-transcriptional regulation | sequester |  | RP11-598D14.1 inhibits IGF2 translation by sequestering IGF2BP1 | down-regulation | EZH2 inhibits RP11-598D14.1 expression by inducing  histone 3 lysine 27 trimethylation |
| TCAM1P-004[30] | protein | IGF2BP1 | mRNA post-transcriptional regulation | sequester |  | TCAM1P-004 inhibits IGF2 translation by sequestering IGF2BP1 | down-regulation | EZH2 inhibits TCAM1P-004 expression by inducing  histone 3 lysine 27 trimethylation |
| DANCR [31] | mRNA | β-catenin mRNA | mRNA post-transcriptional regulation | sequester | cytoplasm | DANCR increases β-catenin mRNA by competitively binding to its 3'UTR by to block miR-214/199a/320a | up-regulation |  |
| HULC [32] | mRNA | Clock circadian regulator (CLOCK) mRNA | mRNA post-transcriptional regulation | sequester |  | HULC increases CLOCK stability by binding its 5'UTR | up-regulation |  |
| ICR [33] | mRNA | ICAM-1 mRNA | mRNA post-transcriptional regulation | sequester |  | ICR increases the stability of ICAM-1 mRNA by binding with it | up-regulation | Nanog promotes ICR transcription by binding to the ICR promoter |
| lncRNA-ATB [34] | mRNA | IL-11 mRNA | mRNA post-transcriptional regulation | sequester | cytoplasm | lncRNA-ATB increases IL-11 mRNA stability by binding with it | up-regulation |  |
| PXN-AS1-L [35] | mRNA | PXN mRNA | mRNA post-transcriptional regulation | sequester | cytoplasm | MBNL3 induces lncRNA-PXN-AS1-L exon 4 inclusion, exon4 promotes PXN mRNA stability by binding its 3'-UTR | up-regulation | MBNL3-mediated alternative splicing |
| PXN-AS1-S [35] | mRNA | PXN mRNA | mRNA post-transcriptional regulation | sequester | cytoplasm | lncRNA-PXN-AS1-S cannot promote PXN mRNA translation | down- regulation | MBNL4-mediated alternative splicing |
| lncARSR [36] | mRNA | PTEN mRNA | mRNA post-transcriptional regulation |  | cytoplasm | the interaction between lncARSR and PTEN mRNA promotes PTEN mRNA degradation by an unknown mechanism | up-regulation |  |
| RAD51-AS1[37] | mRNA | RAD51  mRNA | mRNA post-transcriptional regulation | sequester |  | RAD51-AS1 binds to RAD51  mRNA to inhibit its translation |  | Melatonin induces expression of RAD51-AS1 |
| AF113014 [38] | miRNA | miR-20a | mRNA post-transcriptional regulation | sequester |  | AF113014 promotes Egr2 translation by sequestering miR-20a | down regulated |  |
| ASLNC02525 [39] | miRNA | hsa-mi489-3p | mRNA post-transcriptional regulation | sequester |  | ASLNC02525 promotes twist1 translation by sequestering hsa-mi489-3p | up-regulation |  |
| CCAT1 [40] | miRNA | let-7 | mRNA post-transcriptional regulation | sequester |  | CCAT1 promotes HMGA2 and c-Myc translation by sequestering let-7 | up-regulation |  |
| CCAT2 [41] | miRNA | miR-34a | mRNA post-transcriptional regulation | sequester | cytoplasm | CCAT2 promotes FOXM1 translation by sequestering miR-134a | up-regulation | FOXM1 promotes CCAT2 transcription by binding its promoter |
| CDKN2B-AS1 [42] | miRNA | let-7c-5p | mRNA post-transcriptional regulation | sequester |  | CDKN2B-AS1 promotes nucleosome assembly protein 1 like 1 (NAP1L1) translation by sequestering let-7c-5p | up-regulation |  |
| CRNDE [43] | miRNA | miR-384 | mRNA post-transcriptional regulation | sequester |  | CRNDE promotes NF-κB and p-AKT translation by sequestering miR-384 | up-regulation |  |
| DLX6-AS1 [44] | miRNA | miR-203a | mRNA post-transcriptional regulation | sequester |  | DLX6-AS1 promotes MMP-2 translation by sequestering miR-203a | up-regulation |  |
| DSCR8 [45] | miRNA | miR-485-5p | mRNA post-transcriptional regulation | sequester | nucleus and cytoplasm | DSCR8 promotes Frizzled-7 translation by sequestering miR-485-5p | up-regulation |  |
| FABP5P3 [46] | miRNA | miR-589-5p | mRNA post-transcriptional regulation | sequester |  | FABP5P3 promotes ZMYND19 translation by sequestering miR-589-5p | up-regulation |  |
| FAL1 [47] | miRNA | miR-1236 | mRNA post-transcriptional regulation | sequester |  | FAL1 promotes AFP and ZEB1 translation by sequestering miR-1236 | up-regulation |  |
| FLVCR1-AS1 [48] | miRNA | miR-513c | mRNA post-transcriptional regulation | sequester |  | FLVCR1-AS1 promotes MET translation by sequestering miR-513c | up-regulation |  |
| H19 [49] | miRNA | miR-193a-3p | mRNA post-transcriptional regulation | sequester |  | H19 promotes PSEN1 translation by sequestering miR-193a-3p | up-regulation |  |
| HBx-LINE1[50] | miRNA | miR-122 | mRNA post-transcriptional regulation | sequester |  | HBx-LINE1 promotes miR-122 target genes translation by sequestering miR-122 | up-regulation |  |
| HNF1A-AS1 [51] | miRNA | hsa-miR-30b-5p | mRNA post-transcriptional regulation | sequester |  | HNF1A-AS1 promotes Bcl-2 and ATG5 translation by sequestering hsa-miR-30b-5p | up-regulation |  |
| HOXD-AS1 [52] | miRNA | miR-19a | mRNA post-transcriptional regulation | sequester | nucleus and cytoplasm | HOXD-AS1 promotes Rho GTPase activating protein 11A translation by sequestering miR-19a | up-regulation |  |
| HOXD-AS1 [53] | miRNA | miR-130a-3p | mRNA post-transcriptional regulation | sequester | cytoplasm | HOXD-AS1 promotes SOX4 translation by sequestering miR-130a-3p | up-regulation | STAT3 promotes HOXD-AS1 transcription by binding its promoter |
| HULC [54] | miRNA | miR-372 | mRNA post-transcriptional regulation | sequester | cytoplasm | HULC promotes PRKACB translation by sequestering miR-372 | up-regulation | phospho-CREB promotes HULC transcription by binding to its promoter |
| HULC [55] | miRNA | miR-186 | mRNA post-transcriptional regulation | sequester |  | HULC promotes HMGA2 translation by sequestering miR-186 | up-regulation |  |
| KCNQ1OT1 [56] | miRNA | miR-504 | mRNA post-transcriptional regulation | sequester |  | KCNQ1OT1 promotes cyclin-dependent kinase 16 translation by sequestering miR-504 | up-regulation |  |
| KRAL [57] | miRNA | miR-141 | mRNA post-transcriptional regulation | sequester | cytoplasm | KRAL promotes Keap1 translation by sequestering miR-141 | down- regulation in 5-fluorouracilresistant HCC cells |  |
| linc00176 [58] | miRNA | miR-9 and miR-185 | mRNA post-transcriptional regulation | sequester | cytoplasm | linc00176 promotes miR-9 and miR-185 target genes translation by sequestering miR-9 and miR-185 | up-regulation | Linc00176 is regulated by Myc/Max and AP-4 transcription factors |
| Linc00974 [59] | miRNA | miR-642 | mRNA post-transcriptional regulation | sequester | cytoplasm | Linc00974 promotes KRT19 translation by sequestering miR-642 | up-regulation |  |
| LINC01287 [60] | miRNA | miR-298 | mRNA post-transcriptional regulation | sequester | cytoplasm | LINC01287 promotes STAT3 translation by sequestering miR-298 | up-regulation | STAT3 elevates LINC01287 expression via c-Jun, which binds to the LINC01287 promoter. |
| linc-USP16 [61] | miRNA | miR-21 and miR-590-5p | mRNA post-transcriptional regulation | sequester | cytoplasm | linc-USP16 promotes PTEN translation by sequestering miR-21 and miR-590-5p | down- regulation |  |
| lnc-FTX [62] | miRNA | miR-374a | mRNA post-transcriptional regulation | sequester | nucleus and cytoplasm | lnc-FTX promotes WIF1, PTEN and WNT5A translation by sequestering miR-374a | down- regulation |  |
| lncRNA-657 [63] | miRNA | miR-106a-5p | mRNA post-transcriptional regulation | sequester |  | lncRNA-657 promotes PTEN translation by sequestering miR-106a-5p | down- regulation |  |
| lncRNA-ATB [34] | miRNA | miR-200s | mRNA post-transcriptional regulation | sequester | cytoplasm | lncRNA-ATB promotes ZEB1 and ZEB2 translation by sequestering miR-200s | up-regulation |  |
| lncRNA-ATB [64] | miRNA | miR-200 | mRNA post-transcriptional regulation | sequester |  | lncRNA-ATB promotes β-catenin translation by sequestering miR-200 | up-regulation |  |
| lncRNA-HCAL [65] | miRNA | miR-15a and miR-196a/b | mRNA post-transcriptional regulation | sequester |  | lncRNA-HCAL promotes LAPTM4B translation by sequestering miR-15a and miR-196a/b | up-regulation |  |
| lncRNA–MUF [66] | miRNA | miR-34a | mRNA post-transcriptional regulation | sequester | cytoplasm | lncRNA-MUF promotes Snail1 translation by sequestering miR-34a | up-regulation |  |
| MALAT1 [67] | miRNA | miR-216b | mRNA post-transcriptional regulation | sequester |  | MALAT1 promotes insulin-like growth factor 2 mRNA binding protein 2 translation by sequestering miR-216b | up-regulation | HIF-2a promotes MALAT1 upregulation in HCC cells |
| MALAT1 [68] | miRNA | miR-143-3p | mRNA post-transcriptional regulation | sequester |  | MALAT1 promotes ZEB1 translation by sequestering miR-143-3p | up-regulation |  |
| MCM3AP-AS1 [69] | miRNA | miR-194-5p | mRNA post-transcriptional regulation | sequester |  | MCM3AP-AS1 promotes forkhead box A1 translation by sequestering miR-194-5p | up-regulation |  |
| MIR31HG[70] | miRNA | miR-575 | mRNA post-transcriptional regulation | sequester | cytoplasm | MIR31HG promotes tumorigenicity 7 like translation by sequestering miiR-575 | down-regulation |  |
| n335586 [71] | miRNA | miR-924 | mRNA post-transcriptional regulation | sequester | cytoplasm | n335586 promotes CKMT1A translation by sequestering miR-924 | up-regulation | lncRNA n335586 is induced by HBV |
| NEAT1 [72] | miRNA | miR-485 | mRNA post-transcriptional regulation | sequester |  | NEAT1 promotes STAT3 translation by sequestering miR-485 | up-regulation |  |
| NEAT1 [73] | miRNA | miR-486 | mRNA post-transcriptional regulation | sequester |  | NEAT1 promotes adipose triglyceride lipase translation by sequestering miR-124-3p | up-regulation |  |
| NEAT1 [74] | miRNA | hsa-miR-139-5p | mRNA post-transcriptional regulation | sequester |  | NEAT1 promotes TGF-β1 translation by sequestering hsa-miR-139-5p | up-regulation |  |
| NR2F1-AS1 [75] | miRNA | miR-363 | mRNA post-transcriptional regulation | sequester |  | NR2F1-AS1 promotes ABCC1 translation by sequestering miR-363 | up-regulation |  |
| NRAL [76] | miRNA | miR-340-5p | mRNA post-transcriptional regulation | sequester |  | NRAL promotes Nrf2 translation by sequestering miR-340-5p | up-regulation |  |
| PCAT-1 [77] | miRNA | miR-129-5p | mRNA post-transcriptional regulation | sequester |  | PCAT-1 promotes HMGB1 translation by sequestering miR-129-5p | up-regulation |  |
| SBF2-AS1 [78] | miRNA | miR-140-5p | mRNA post-transcriptional regulation | sequester |  | SBF2-AS1 promotes transforming growth factor beta receptor 1 (TGFBR1) translation by sequestering miR-140-5p | up-regulation |  |
| SNHG12 [79] | miRNA | miR-199a/b-5p | mRNA post-transcriptional regulation | sequester | cytoplasm | SNHG12 promotes MLK3 translation by sequestering miR-199a/b-5p | up-regulation |  |
| SNHG3 [80] | miRNA | miR‐128 | mRNA post-transcriptional regulation | sequester |  | SNHG3 promotes CD151 translation by sequestering miR-128 | down-regulation |  |
| SNHG5 [81] | miRNA | miR-26a-5p | mRNA post-transcriptional regulation | sequester | cytoplasm | SNHG5 promotes GSK3β translation by sequestering miR-26a-5p | up-regulation |  |
| SNHG6 [82] | miRNA | miR-1297 | mRNA post-transcriptional regulation | sequester | cytoplasm | SNHG6 promotes MAT2A translation by sequestering miR-1297 | up-regulation |  |
| SNHG6-003 [83] | miRNA | miR-26a/b | mRNA post-transcriptional regulation | sequester | cytoplasm | SNHG6-003 promotes transforming growth factor-beta-activated kinase 1 (TAK1) translation by sequestering miR-26a/b | up-regulation |  |
| TP73-AS1 [84] | miRNA | miR-200a | mRNA post-transcriptional regulation | sequester |  | TP73-AS1 promotes HMGB1 translation by sequestering miR-200a | up-regulation |  |
| TUG1 [85] | miRNA | miR-144 | mRNA post-transcriptional regulation | sequester |  | TUG1 promotes JAK2 translation by sequestering miR-144 | up-regulation |  |
| TUG1 [86] | miRNA | miR-142-3p | mRNA post-transcriptional regulation | sequester |  | TUG1 promotes ZEB1 translation by sequestering miR-142-3p | up-regulation |  |
| UCA1 [87] | miRNA | miR-203 | mRNA post-transcriptional regulation | sequester |  | UCA1 promotes Snail2 translation by sequestering miR-203 | up-regulation |  |
| Unigene56159 [88] | miRNA | miR-140-5p | mRNA post-transcriptional regulation | sequester |  | Unigene56159 promotes Slug translation by sequestering miR-140-5p | up-regulation |  |
| XIST [89] | miRNA | miR-181a | mRNA post-transcriptional regulation | sequester |  | XIST promotes PTEN translation by sequestering miR-181a | down-regulation |  |
| XIST [90] | miRNA | miR-194-5p | mRNA post-transcriptional regulation | sequester |  | XIST promotes MAPK1 translation by sequestering miR-194-5p | up-regulation |  |
| ZFAS1 [91] | miRNA | miR-150 | mRNA post-transcriptional regulation | sequester |  | ZFAS1 promotes ZEB1, MMP14, and MMP16 by sequestering miR-150a/b | up-regulation |  |
| HOTAIR [92] | protein | suppressor of zeste 12 homolog (SUZ12) and DDX5 | protein degradation | scaffold | nucleus and cytoplasm | Mex3b displaces DDX5 from HOTAIR, ubiquitinates SUZ12, and induces SUZ12 degradation | up-regulation |  |
| LNC473 [93] | protein | survivin and USP9X | protein degradation | scaffold |  | LNC473 interacts with survivin and USP9X to inhibit the ubiquitination level of survivin | up-regulation |  |
| LINC01138 [94] | protein | PRMT5 | protein degradation | sequester | nucleus and cytoplasm | LINC01138 binds to PRMT5, blocking its ubiquitination by CHIP E3 | up-regulation | gene copy-number alteration |
| lnc-EGFR [95] | protein | epidermal growth factor receptor | protein degradation | sequester | cytoplasm | lnc-EGFR binds to EGFR, blocking its ubiquitination by c-CBL | up-regulation |  |
| uc.134 [96] | protein | E3 ubiquitin ligase CUL4A | protein degradation | sequester | cytoplasm | uc.134 inhibits the CUL4A translocation from the nucleus to the cytoplasm and disrupts ubiquitination of LATS | down- regulation |  |
| lncRNA-hPVT1 [97] | protein | NOP2 | protein degradation |  | nucleus | the association between hPVT1 and NOP2 enhances NOP2 stability | up- regulation | TGF-β1 can up-regulate the expression of lncRNA-hPVT1 |
| lncRNA-LET [98] | protein | NF90 | protein degradation |  | cytoplasm | the interaction between lncNRA-LET and NF90 increases the ubiquitination level of NF90 by an unknown mechanism | down- regulation | HDAC3 reduces histone H3 and H4 acetylation levels in the lncRNA-LET promoter region |
| lncRNA-PRAL [99] | protein | HSP90 and p53 | protein degradation |  | nucleus and cytoplasm | lncRNA-PRAL enhance the interaction between p53 and HSP90, which inhibits MDM2 induced p53 ubiquitination and degradation by directly binding with HSP90 | down-regulation | gene copy-number alteration |
| lnc-β-Catm [100] | protein | β-catenin and EZH2 | protein methylation | scaffold | nucleus | lnc-β-Catm interacts with β-catenin and EZH2 simultaneously, thereby promoting the methylation of β-catenin | up-regulation (in CSCs) | gene copy-number alteration |
| HULC [101] | protein | Y-box binding protein 1(YB-1) and extracellular signal-regulated kinase | protein phosphorylation | scaffold | cytoplasm | HULC interacts with YB-1 and extracellular signal-regulated kinase simultaneously, thereby promoting the phosphorylation of YB1 | up-regulation | IGF2 mRNA-binding proteins recruits the CCR4-NOT complex to promote the degradation of the lncRNA HULC [102] |
| HANR [103] | protein | GSK3β interaction protein | protein phosphorylation | sequester | cytoplasm | the interaction between HANR and GSKIP inhibits the phosphorylation level of GSK3β | up-regulation |  |
| TSLNC8 [104] | protein | STAT3 or TKT | protein phosphorylation | sequester | nucleus:75% | TSLNC8 sequesters STAT3 or TKT to disrupt the interaction between STAT3 and TKT | down-regulation | chromosome loss/copy number loss |
| HNF1A-AS1 [105] {Ding, 2018 #276} | protein | SHP-1 | protein phosphorylation |  |  | HNF1A-AS1 promotes the phosphatase activity of SHP-1 by binding with it. | down-regulation | HNF1α enhances HNF1A-AS1 transcription by directly binding to its promoter |
| lncRNA–MUF [66] | protein | Annexin A2 and glycogen synthase kinase 3b | protein complex modulation | scaffold | cytoplasm | lncRNA-MUF bridges the interaction between GSK-3β and ANXA2, thereby disrupting the formation of GSK-3β/β-catenin complex | up-regulation |  |
| Linc00210 [106] | protein | CTNNBIP1 | protein complex modulation | sequester | nucleus | Linc00210 promotes interaction of β-catenin and TCF/LEF components by sequestering CTNNBIP1 | up-regulation |  |
| lncBRM [107] | protein | BRM | protein complex modulation | sequester | nucleus | lncBRM binds to BRM, which induces BRG1/BRM switch in BAF complex and increases BRG1-embedded BAF complex level | up-regulation |  |
| lnc-FTX [62] | protein | DNA replication licensing factor MCM2 | protein complex modulation | sequester | nucleus and cytoplasm | lnc-FTC might disrupt the formation of prereplicative complex by precluding MCM2 loading onto chromatin [108] | down-regulation |  |
| lnc-Tim3 [109] | protein | Tim-3 | protein complex modulation | sequester |  | the interaction between lnc-Tim3 and Tim-3 releases Bat3 from Tim-3 and free to form a complex with p300 | up-regulation |  |

**References for tableS1**

1. Huang, M.D., et al., *Long non-coding RNA ANRIL is upregulated in hepatocellular carcinoma and regulates cell apoptosis by epigenetic silencing of KLF2.* J Hematol Oncol, 2015. **8**: p. 50.

2. Jin, L., et al., *LncRNA GHET1 predicts poor prognosis in hepatocellular carcinoma and promotes cell proliferation by silencing KLF2.* J Cell Physiol, 2018. **233**(6): p. 4726-4734.

3. Sui, C.J., et al., *Long noncoding RNA GIHCG promotes hepatocellular carcinoma progression through epigenetically regulating miR-200b/a/429.* J Mol Med (Berl), 2016. **94**(11): p. 1281-1296.

4. Zhu, X., et al., *Long noncoding RNA glypican 3 (GPC3) antisense transcript 1 promotes hepatocellular carcinoma progression via epigenetically activating GPC3.* FEBS J., 2016. **283**(20): p. 3739-3754.

5. Zhang, L., et al., *Epigenetic activation of the MiR-200 family contributes to H19-mediated metastasis suppression in hepatocellular carcinoma.* Carcinogenesis, 2013. **34**(3): p. 577-86.

6. Zhang, J., et al., *A novel TGF-β and H19 signaling axis in tumor-initiating hepatocytes that regulates hepatic carcinogenesis.* Hepatology, 2018.

7. Wang, C., et al., *Long non-coding RNA HNF1A-AS1 promotes hepatocellular carcinoma cell proliferation by repressing NKD1 and P21 expression.* Biomed Pharmacother, 2017. **89**: p. 926-932.

8. Tang, J., et al., *Bidirectional transcription of Linc00441 and RB1 via H3K27 modification-dependent way promotes hepatocellular carcinoma.* Cell Death Dis, 2017. **8**(3): p. e2675.

9. Zeng, B., et al., *Upregulation of LncDQ is Associated with Poor Prognosis and Promotes Tumor Progression via Epigenetic Regulation of the EMT Pathway in HCC.* Cell Physiol Biochem, 2018. **46**(3): p. 1122-1133.

10. Yang, F., et al., *Long noncoding RNA high expression in hepatocellular carcinoma facilitates tumor growth through enhancer of zeste homolog 2 in humans.* Hepatology, 2011. **54**(5): p. 1679-89.

11. Wang, Y., et al., *The long noncoding RNA lncTCF7 promotes self-renewal of human liver cancer stem cells through activation of Wnt signaling.* Cell Stem Cell, 2015. **16**(4): p. 413-25.

12. Wu, J., et al., *Long noncoding RNA lncTCF7, induced by IL-6/STAT3 transactivation, promotes hepatocellular carcinoma aggressiveness through epithelial-mesenchymal transition.* J Exp Clin Cancer Res, 2015. **34**: p. 116.

13. Wei, C., et al., *LncRNA SOX21-AS1 is associated with progression of hepatocellular carcinoma and predicts prognosis through epigenetically silencing p21.* Biomed Pharmacother, 2018. **104**: p. 137-144.

14. Zhou, M., X.Y. Zhang, and X. Yu, *Overexpression of the long non-coding RNA SPRY4-IT1 promotes tumor cell proliferation and invasion by activating EZH2 in hepatocellular carcinoma.* Biomed Pharmacother, 2017. **85**: p. 348-354.

15. Huang, M.D., et al., *Long non-coding RNA TUG1 is up-regulated in hepatocellular carcinoma and promotes cell growth and apoptosis by epigenetically silencing of KLF2.* Mol Cancer, 2015. **14**: p. 165.

16. Battistelli, C., et al., *The Snail repressor recruits EZH2 to specific genomic sites through the enrollment of the lncRNA HOTAIR in epithelial-to-mesenchymal transition.* Oncogene, 2017. **36**(7): p. 942-955.

17. Xu, D., et al., *Long noncoding RNAs associated with liver regeneration 1 accelerates hepatocyte proliferation during liver regeneration by activating Wnt/beta-catenin signaling.* Hepatology, 2013. **58**(2): p. 739-51.

18. Chen, Z.Z., et al., *LncSox4 promotes the self-renewal of liver tumour-initiating cells through Stat3-mediated Sox4 expression.* Nat Commun, 2016. **7**: p. 12598.

19. Chen, B., *A novel long noncoding RNA lncWDR26 suppresses the growth and metastasis of hepatocellular carcinoma cells through interaction with SIX3.* Am J Cancer Res, 2018. **8**(4): p. 688-698.

20. Sun, Q.M., et al., *Long non-coding RNA00607 as a tumor suppressor by modulating NF-kappaB p65/p53 signaling axis in hepatocellular carcinoma.* Carcinogenesis, 2018.

21. Ding, L.J., et al., *Long Noncoding RNA lncCAMTA1 Promotes Proliferation and Cancer Stem Cell-Like Properties of Liver Cancer by Inhibiting CAMTA1.* Int J Mol Sci, 2016. **17**(10).

22. Wang, X., et al., *Long non-coding RNA DILC regulates liver cancer stem cells via IL-6/STAT3 axis.* J Hepatol, 2016. **64**(6): p. 1283-94.

23. Liu, N., et al., *Hepatitis B virus-upregulated lnc-HUR1 promotes cell proliferation and tumorigenesis by blocking p53 activity.* Hepatology, 2018.

24. Liang, W.C., et al., *LncRNA-NEF antagonized epithelial to mesenchymal transition and cancer metastasis via cis-regulating FOXA2 and inactivating Wnt/beta-catenin signaling.* Oncogene, 2018. **37**(11): p. 1445-1456.

25. Lv, L., et al., *WT1-AS promotes cell apoptosis in hepatocellular carcinoma through down-regulating of WT1.* J Exp Clin Cancer Res, 2015. **34**: p. 119.

26. Klingenberg, M., et al., *The lncRNA CASC9 and RNA binding protein HNRNPL form a complex and co-regulate genes linked to AKT signaling.* Hepatology, 2018.

27. Cao, C., et al., *The long intergenic noncoding RNA UFC1, a target of MicroRNA 34a, interacts with the mRNA stabilizing protein HuR to increase levels of beta-catenin in HCC cells.* Gastroenterology, 2015. **148**(2): p. 415-26 e18.

28. Wang, Y.L., et al., *Lnc-UCID promotes G1/S transition and hepatoma growth by preventing DHX9-mediated CDK6 downregulation.* Hepatology, 2019.

29. Zhang, D.Y., et al., *Identification and Functional Characterization of Long Non-coding RNA MIR22HG as a Tumor Suppressor for Hepatocellular Carcinoma.* Theranostics, 2018. **8**(14): p. 3751-3765.

30. F, X., et al., *Genome-wide screening and functional analysis identifies tumor suppressor long non-coding RNAs epigenetically silenced in hepatocellular carcinoma.* Cancer research, 2019. **undefined**(undefined): p. undefined.

31. Yuan, S.X., et al., *Long noncoding RNA DANCR increases stemness features of hepatocellular carcinoma by derepression of CTNNB1.* Hepatology, 2016. **63**(2): p. 499-511.

32. Cui, M., et al., *A long noncoding RNA perturbs the circadian rhythm of hepatoma cells to facilitate hepatocarcinogenesis.* Neoplasia, 2015. **17**(1): p. 79-88.

33. Guo, W., et al., *ICAM-1-Related Noncoding RNA in Cancer Stem Cells Maintains ICAM-1 Expression in Hepatocellular Carcinoma.* Clin Cancer Res, 2016. **22**(8): p. 2041-50.

34. Yuan, J.H., et al., *A long noncoding RNA activated by TGF-beta promotes the invasion-metastasis cascade in hepatocellular carcinoma.* Cancer Cell, 2014. **25**(5): p. 666-81.

35. Yuan, J.H., et al., *The MBNL3 splicing factor promotes hepatocellular carcinoma by increasing PXN expression through the alternative splicing of lncRNA-PXN-AS1.* Nat Cell Biol, 2017. **19**(7): p. 820-832.

36. Li, Y., et al., *Long Noncoding RNA lncARSR Promotes Doxorubicin Resistance in Hepatocellular Carcinoma via Modulating PTEN-PI3K/Akt Pathway.* J. Cell. Biochem., 2017. **118**(12): p. 4498-4507.

37. Chen, C.C., et al., *Melatonin Sensitizes Hepatocellular Carcinoma Cells to Chemotherapy Through Long Non-Coding RNA RAD51-AS1-Mediated Suppression of DNA Repair.* Cancers (Basel), 2018. **10**(9).

38. Zeng, T., et al., *LncRNA-AF113014 promotes the expression of Egr2 by interaction with miR-20a to inhibit proliferation of hepatocellular carcinoma cells.* PLoS One, 2017. **12**(5): p. e0177843.

39. Chen, Z., D. Xu, and T. Zhang, *Inhibition of proliferation and invasion of hepatocellular carcinoma cells by lncRNA-ASLNC02525 silencing and the mechanism.* Int J Oncol, 2017. **51**(3): p. 851-858.

40. Deng, L., et al., *Long noncoding RNA CCAT1 promotes hepatocellular carcinoma progression by functioning as let-7 sponge.* J Exp Clin Cancer Res, 2015. **34**: p. 18.

41. Chen, F., et al., *A positive feedback loop of long noncoding RNA CCAT2 and FOXM1 promotes hepatocellular carcinoma growth.* Am J Cancer Res, 2017. **7**(7): p. 1423-1434.

42. Huang, Y., et al., *LncRNA CDKN2B-AS1 promotes tumor growth and metastasis of human hepatocellular carcinoma by targeting let-7c-5p/NAP1L1 axis.* Cancer Lett, 2018.

43. Chen, Z., et al., *LncRNA CRNDE promotes hepatic carcinoma cell proliferation, migration and invasion by suppressing miR-384.* Am J Cancer Res, 2016. **6**(10): p. 2299-2309.

44. Zhang, L., et al., *Long non-coding RNA DLX6-AS1 aggravates hepatocellular carcinoma carcinogenesis by modulating miR-203a/MMP-2 pathway.* Biomed Pharmacother, 2017. **96**: p. 884-891.

45. Wang, Y., et al., *Long non-coding RNA DSCR8 acts as a molecular sponge for miR-485-5p to activate Wnt/beta-catenin signal pathway in hepatocellular carcinoma.* Cell Death Dis, 2018. **9**(9): p. 851.

46. Zhu, Q., et al., *LncRNA FABP5P3/miR-589-5p/ZMYND19 axis contributes to hepatocellular carcinoma cell proliferation, migration and invasion.* Biochem. Biophys. Res. Commun., 2018. **498**(3): p. 551-558.

47. Li, B., et al., *LncRNA FAL1 promotes cell proliferation and migration by acting as a CeRNA of miR-1236 in hepatocellular carcinoma cells.* Life Sci, 2018. **197**: p. 122-129.

48. Zhang, K., et al., *LncRNA FLVCR1-AS1 acts as miR-513c sponge to modulate cancer cell proliferation, migration, and invasion in hepatocellular carcinoma.* J Cell Biochem, 2018. **119**(7): p. 6045-6056.

49. Ma, H., et al., *The LncRNA H19/miR-193a-3p axis modifies the radio-resistance and chemotherapeutic tolerance of hepatocellular carcinoma cells by targeting PSEN1.* J. Cell. Biochem., 2018.

50. N, W., et al., *Hepatitis B virus-human chimeric transcript HBx-LINE1 promotes hepatic injury via sequestering cellular microRNA-122.%A Liang HW.* Journal of hepatology, 2016. **64**(2): p. 278-291.

51. Liu, Z., et al., *Long non-coding RNA HNF1A-AS1 functioned as an oncogene and autophagy promoter in hepatocellular carcinoma through sponging hsa-miR-30b-5p.* Biochem Biophys Res Commun, 2016. **473**(4): p. 1268-1275.

52. Lu, S., et al., *The noncoding RNA HOXD-AS1 is a critical regulator of the metastasis and apoptosis phenotype in human hepatocellular carcinoma.* Mol Cancer, 2017. **16**(1): p. 125.

53. Wang, H., et al., *STAT3-mediated upregulation of lncRNA HOXD-AS1 as a ceRNA facilitates liver cancer metastasis by regulating SOX4.* Molecular Cancer, 2017. **16**(1).

54. Wang, J., et al., *CREB up-regulates long non-coding RNA, HULC expression through interaction with microRNA-372 in liver cancer.* Nucleic Acids Res, 2010. **38**(16): p. 5366-83.

55. Wang, Y., et al., *The long noncoding RNA HULC promotes liver cancer by increasing the expression of the HMGA2 oncogene via sequestration of the microRNA-186.* J Biol Chem, 2017. **292**(37): p. 15395-15407.

56. Li, C., et al., *Long non-coding RNA KCNQ1OT1 mediates the growth of hepatocellular carcinoma by functioning as a competing endogenous RNA of miR-504.* Int J Oncol, 2018.

57. Wu, L., et al., *lncRNA KRAL reverses 5-fluorouracil resistance in hepatocellular carcinoma cells by acting as a ceRNA against miR-141.* Cell Commun Signal, 2018. **16**(1): p. 47.

58. Tran, D.D.H., et al., *Myc target gene, long intergenic noncoding RNA, Linc00176 in hepatocellular carcinoma regulates cell cycle and cell survival by titrating tumor suppressor microRNAs.* Oncogene, 2018. **37**(1): p. 75-85.

59. Tang, J., et al., *A novel biomarker Linc00974 interacting with KRT19 promotes proliferation and metastasis in hepatocellular carcinoma.* Cell Death Dis, 2014. **5**: p. e1549.

60. Mo, Y., et al., *LINC01287/miR-298/STAT3 feedback loop regulates growth and the epithelial-to-mesenchymal transition phenotype in hepatocellular carcinoma cells.* J Exp Clin Cancer Res, 2018. **37**(1): p. 149.

61. Sui, J., et al., *Long Non-Coding RNA Linc-USP16 Functions As a Tumour Suppressor in Hepatocellular Carcinoma by Regulating PTEN Expression.* Cell Physiol Biochem, 2017. **44**(3): p. 1188-1198.

62. Liu, F., et al., *Long noncoding RNA FTX inhibits hepatocellular carcinoma proliferation and metastasis by binding MCM2 and miR-374a.* Oncogene, 2016. **35**(41): p. 5422-5434.

63. Hu, B., et al., *Long non-coding RNA 657 suppresses hepatocellular carcinoma cell growth by acting as a molecular sponge of miR-106a-5p to regulate PTEN expression.* Int J Biochem Cell Biol, 2017. **92**: p. 34-42.

64. Fu, N., et al., *LncRNA-ATB/microRNA-200a/β-catenin regulatory axis involved in the progression of HCV-related hepatic fibrosis.* Gene, 2017. **618**: p. 1-7.

65. Xie, C.R., et al., *Long Noncoding RNA HCAL Facilitates the Growth and Metastasis of Hepatocellular Carcinoma by Acting as a ceRNA of LAPTM4B.* Mol Ther Nucleic Acids, 2017. **9**: p. 440-451.

66. Yan, X., et al., *Mesenchymal Stem Cells Promote Hepatocarcinogenesis via lncRNA-MUF Interaction with ANXA2 and miR-34a.* Cancer Res, 2017. **77**(23): p. 6704-6716.

67. Yuan, P., et al., *The HIF-2alpha-MALAT1-miR-216b axis regulates multi-drug resistance of hepatocellular carcinoma cells via modulating autophagy.* Biochem Biophys Res Commun, 2016. **478**(3): p. 1067-73.

68. Chen, L., et al., *Long Non-Coding RNA MALAT1 Regulates ZEB1 Expression by Sponging miR-143-3p and Promotes Hepatocellular Carcinoma Progression.* J Cell Biochem, 2017. **118**(12): p. 4836-4843.

69. Wang, Y., et al., *A novel lncRNA MCM3AP-AS1 promotes the growth of hepatocellular carcinoma by targeting miR-194-5p/FOXA1 axis.* Mol Cancer, 2019. **18**(1): p. 28.

70. Yan, S., et al., *Long noncoding RNA MIR31HG inhibits hepatocellular carcinoma proliferation and metastasis by sponging microRNA-575 to modulate ST7L expression.* J Exp Clin Cancer Res, 2018. **37**(1): p. 214.

71. Fan, H., et al., *LncRNA n335586/miR-924/CKMT1A axis contributes to cell migration and invasion in hepatocellular carcinoma cells.* Cancer Letters, 2018. **429**: p. 89-99.

72. Zhang, X.N., J. Zhou, and X.J. Lu, *The long noncoding RNA NEAT1 contributes to hepatocellular carcinoma development by sponging miR-485 and enhancing the expression of the STAT3.* J Cell Physiol, 2018. **233**(9): p. 6733-6741.

73. Liu, X., et al., *Long non-coding RNA NEAT1-modulated abnormal lipolysis via ATGL drives hepatocellular carcinoma proliferation.* Mol Cancer, 2018. **17**(1): p. 90.

74. Tu, J., et al., *NEAT1 upregulates TGF-beta1 to induce hepatocellular carcinoma progression by sponging hsa-mir-139-5p.* J Cell Physiol, 2018.

75. Huang, H., et al., *LncRNA NR2F1-AS1 regulates hepatocellular carcinoma oxaliplatin resistance by targeting ABCC1 via miR-363.* J Cell Mol Med, 2018. **22**(6): p. 3238-3245.

76. Wu, L.L., et al., *NRAL mediates cisplatin resistance in hepatocellular carcinoma via miR-340-5p/Nrf2 axis.* J Cell Commun Signal, 2018.

77. Zhang, D., et al., *Long noncoding RNA PCAT-1 promotes invasion and metastasis via the miR-129-5p-HMGB1 signaling pathway in hepatocellular carcinoma.* Biomed Pharmacother, 2017. **95**: p. 1187-1193.

78. Li, Y., et al., *Long non-coding RNA SBF2-AS1 promotes hepatocellular carcinoma progression through regulation of miR-140-5p-TGFBR1 pathway.* Biochem Biophys Res Commun, 2018.

79. Lan, T., et al., *Long non-coding RNA small nucleolar RNA host gene 12 (SNHG12) promotes tumorigenesis and metastasis by targeting miR-199a/b-5p in hepatocellular carcinoma.* J Exp Clin Cancer Res, 2017. **36**(1): p. 11.

80. Zhang, P.F., et al., *LncRNA SNHG3 induces EMT and sorafenib resistance by modulating the miR-128/CD151 pathway in hepatocellular carcinoma.* J Cell Physiol, 2018.

81. Li, Y., et al., *Long non-coding RNA SNHG5 promotes human hepatocellular carcinoma progression by regulating miR-26a-5p/GSK3beta signal pathway.* Cell Death Dis, 2018. **9**(9): p. 888.

82. Guo, T., et al., *SNHG6 Acts as a Genome-Wide Hypomethylation Trigger via Coupling of miR-1297–Mediated S-Adenosylmethionine–Dependent Positive Feedback Loops.* Cancer Research, 2018. **78**(14): p. 3849-3864.

83. Cao, C., et al., *The long non-coding RNA, SNHG6-003, functions as a competing endogenous RNA to promote the progression of hepatocellular carcinoma.* Oncogene, 2017. **36**(8): p. 1112-1122.

84. Li, S., et al., *The long non-coding RNA TP73-AS1 modulates HCC cell proliferation through miR-200a-dependent HMGB1/RAGE regulation.* J Exp Clin Cancer Res, 2017. **36**(1): p. 51.

85. Lv, J., et al., *LncRNA TUG1 interacting with miR-144 contributes to proliferation, migration and tumorigenesis through activating the JAK2/STAT3 pathway in hepatocellular carcinoma.* Int. J. Biochem. Cell Biol., 2018. **101**: p. 19-28.

86. He, C., et al., *lncRNA TUG1-Mediated Mir-142-3p Downregulation Contributes to Metastasis and the Epithelial-to-Mesenchymal Transition of Hepatocellular Carcinoma by Targeting ZEB1.* Cell Physiol Biochem, 2018. **48**(5): p. 1928-1941.

87. Xiao, J.N., et al., *Long non-coding RNA UCA1 regulates the expression of Snail2 by miR-203 to promote hepatocellular carcinoma progression.* J Cancer Res Clin Oncol, 2017. **143**(6): p. 981-990.

88. Lv, J., et al., *Long non-coding RNA Unigene56159 promotes epithelial-mesenchymal transition by acting as a ceRNA of miR-140-5p in hepatocellular carcinoma cells.* Cancer Lett, 2016. **382**(2): p. 166-175.

89. Chang, S., et al., *Long non-coding RNA XIST regulates PTEN expression by sponging miR-181a and promotes hepatocellular carcinoma progression.* BMC Cancer, 2017. **17**(1): p. 248.

90. Kong, Q., et al., *LncRNA XIST functions as a molecular sponge of miR-194-5p to regulate MAPK1 expression in hepatocellular carcinoma cell.* J Cell Biochem, 2018. **119**(6): p. 4458-4468.

91. Li, T., et al., *Amplification of Long Noncoding RNA ZFAS1 Promotes Metastasis in Hepatocellular Carcinoma.* Cancer Res, 2015. **75**(15): p. 3181-91.

92. Zhang, H., et al., *RNA helicase DEAD box protein 5 regulates Polycomb repressive complex 2/Hox transcript antisense intergenic RNA function in hepatitis B virus infection and hepatocarcinogenesis.* Hepatology, 2016. **64**(4): p. 1033-48.

93. Chen, H., et al., *Long noncoding RNA LNC473 inhibits the ubiquitination of survivin via association with USP9X and enhances cell proliferation and invasion in hepatocellular carcinoma cells.* Biochem Biophys Res Commun, 2018. **499**(3): p. 702-710.

94. Li, Z., et al., *The LINC01138 drives malignancies via activating arginine methyltransferase 5 in hepatocellular carcinoma.* Nat Commun, 2018. **9**(1): p. 1572.

95. Jiang, R., et al., *The long noncoding RNA lnc-EGFR stimulates T-regulatory cells differentiation thus promoting hepatocellular carcinoma immune evasion.* Nat Commun, 2017. **8**: p. 15129.

96. Ni, W., et al., *A novel lncRNA uc.134 represses hepatocellular carcinoma progression by inhibiting CUL4A-mediated ubiquitination of LATS1.* J Hematol Oncol, 2017. **10**(1): p. 91.

97. Wang, F., et al., *Oncofetal long noncoding RNA PVT1 promotes proliferation and stem cell-like property of hepatocellular carcinoma cells by stabilizing NOP2.* Hepatology, 2014. **60**(4): p. 1278-90.

98. Yang, F., et al., *Repression of the long noncoding RNA-LET by histone deacetylase 3 contributes to hypoxia-mediated metastasis.* Mol Cell, 2013. **49**(6): p. 1083-96.

99. Zhou, C.C., et al., *Systemic genome screening identifies the outcome associated focal loss of long noncoding RNA PRAL in hepatocellular carcinoma.* Hepatology, 2016. **63**(3): p. 850-63.

100. Zhu, P., et al., *lnc-beta-Catm elicits EZH2-dependent beta-catenin stabilization and sustains liver CSC self-renewal.* Nat Struct Mol Biol, 2016. **23**(7): p. 631-9.

101. Li, D., et al., *Long noncoding RNA HULC modulates the phosphorylation of YB-1 through serving as a scaffold of extracellular signal-regulated kinase and YB-1 to enhance hepatocarcinogenesis.* Hepatology, 2017. **65**(5): p. 1612-1627.

102. Hammerle, M., et al., *Posttranscriptional destabilization of the liver-specific long noncoding RNA HULC by the IGF2 mRNA-binding protein 1 (IGF2BP1).* Hepatology, 2013. **58**(5): p. 1703-12.

103. Xiao, J., et al., *LncRNA HANR Promotes Tumorigenesis and Increase of Chemoresistance in Hepatocellular Carcinoma.* Cell Physiol Biochem, 2017. **43**(5): p. 1926-1938.

104. Zhang, J., et al., *Long noncoding RNA TSLNC8 is a tumor suppressor that inactivates the interleukin-6/STAT3 signaling pathway.* Hepatology, 2018. **67**(1): p. 171-187.

105. Ding, C.-H., et al., *The HNF1α-regulated lncRNA HNF1A-AS1 reverses the malignancy of hepatocellular carcinoma by enhancing the phosphatase activity of SHP-1.* Molecular Cancer, 2018. **17**(1).

106. Fu, X., et al., *Linc00210 drives Wnt/beta-catenin signaling activation and liver tumor progression through CTNNBIP1-dependent manner.* Mol Cancer, 2018. **17**(1): p. 73.

107. Zhu, P., et al., *LncBRM initiates YAP1 signalling activation to drive self-renewal of liver cancer stem cells.* Nat Commun, 2016. **7**: p. 13608.

108. Remus, D., et al., *Concerted loading of Mcm2-7 double hexamers around DNA during DNA replication origin licensing.* Cell, 2009. **139**(4): p. 719-30.

109. Ji, J., et al., *Long non-coding RNA Lnc-Tim3 exacerbates CD8 T cell exhaustion via binding to Tim-3 and inducing nuclear translocation of Bat3 in HCC.* Cell Death Dis, 2018. **9**(5): p. 478.
